# Evolving Landscape of Parkinson’s Disease Research: Challenges and Perspectives

**DOI:** 10.1021/acsomega.4c09114

**Published:** 2025-01-08

**Authors:** Rumiana Tenchov, Janet M. Sasso, Qiongqiong Angela Zhou

**Affiliations:** CAS, a division of the American Chemical Society, Columbus, Ohio 43210, United States

## Abstract

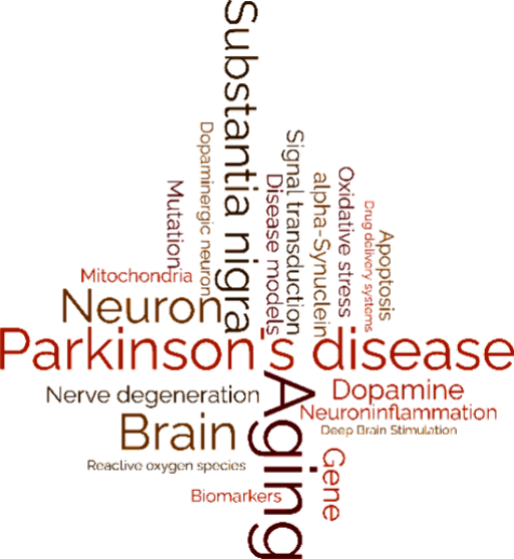

Parkinson’s disease (PD) is a progressive neurodegenerative disorder that primarily affects movement. It occurs due to a gradual deficit of dopamine-producing brain cells, particularly in the substantia nigra. The precise etiology of PD is not fully understood, but it likely involves a combination of genetic and environmental factors. The therapies available at present alleviate symptoms but do not stop the disease’s advancement. Research endeavors are currently directed at inventing disease-controlling therapies that aim at the inherent mechanisms of PD. PD biomarker breakthroughs hold enormous potential: earlier diagnosis, better monitoring, and targeted treatment based on individual response could significantly improve patient outcomes and ease the burden of this disease. PD research is an active and evolving field, focusing on understanding disease mechanisms, identifying biomarkers, developing new treatments, and improving care. In this report, we explore data from the CAS Content Collection to outline the research progress in PD. We analyze the publication landscape to offer perspective into the latest expertise advancements. Key emerging concepts are reviewed and strategies to fight disease evaluated. Pharmacological targets, genetic risk factors, as well as comorbid diseases are explored, and clinical usage of products against PD with their production pipelines and trials for drug repurposing are examined. This review aims to offer a comprehensive overview of the advancing landscape of the current understanding about PD, to define challenges, and to assess growth prospects to stimulate efforts in battling the disease.

## Introduction

Parkinson’s disease (PD) is the second most prevalent neurodegenerative disorder, following Alzheimer’s disease.^1^ Its prevalence increases with age, with most cases occurring in individuals over 60. Prevalence assessments vary globally, but it is usually reported to affect around 1–2% of individuals over the age of 65.^2^ Prevalence rates tend to be higher in industrialized countries compared to developing nations. While most cases of PD are sporadic, a small fraction (∼5–10%) are believed to have a genetic component.^3^ Certain genetic mutations are linked to inherited PD, which may present at an earlier age and have a stronger family history. PD prevalence rates can vary across different regions and countries.^4^ Factors such as environmental exposures, lifestyle habits, and access to healthcare may contribute to these variations, with some studies suggesting higher prevalence rates in certain regions, such as Europe and North America, compared to other parts of the world.^4^ Research into the epidemiology of PD continues to evolve, with efforts to understand regional differences and trends over time.

PD is a progressive neurodegenerative disorder that predominantly impacts movement.^5−7^ It is characterized by the gradual degeneration of dopaminergic neurons in the brain, particularly in a region known as substantia nigra. Dopamine is a neurotransmitter associated with coordinating movement, and its deficiency leads to the characteristic motor symptoms of PD, including tremors, bradykinesia (impairment of voluntary motor control), muscle rigidity, and postural instability.^8^ Additionally, PD can cause a range of nonmotor symptoms, such as cognitive impairment, depression, and sleep disturbances.^9^ The nonmotor symptoms in PD may be caused by a combination of changes to dopaminergic and nondopaminergic neuronal populations.^10−12^ While the exact origin of PD is not fully understood, it is supposed to involve a combination of genetic, environmental, and age-related factors.^5,13^ While there is currently no remedy for PD, treatments are available to manage its symptoms and enhance the quality of life for those affected.

In this review, we outline the research advancement in PD by exploring data from the CAS Content Collection.^14^ The CAS Content Collection is the largest human-curated repository of available scientific information, facilitating thorough analysis of global research. Spanning scientific literature from the past 150 years and over 50 languages, the CAS Content Collection includes data and discoveries from more than 50,000 scientific journals and over 100 patent offices. A key advantage of the CAS Content Collection is that, in addition to regular reference information, it offers human-evaluated data on substances and concepts discussed in scientific publications. The CAS REGISTRY,^15^ an authoritative resource of information on over 250 million unique chemical substances and 70 million protein and nucleic acid sequences, is part of the CAS Content Collection. This collection is widely accessible through CAS solutions such as CAS SciFinder^16^ and CAS STNext.^17^ An inherent limitation of the CAS Content Collection is the natural time lag between research discoveries/inventions happening and them being published, curated, and then analyzed—so, although it provides the best information available, it is necessarily dated.

In this report, we analyze the publication panorama in the field to highlight recent advancements. We discuss key concepts and evaluate strategies to fight the disease. We survey genetic risk factors, pharmacologic targets, and comorbid diseases. We also examine clinical usage of products for PD, including their advancement pipelines and drug repurposing endeavors. This review aims to provide a broad overview of the current PD expertise, identify challenges, and highlight growth opportunities. Its novelty and merit arise from the extensive, wide-ranging coverage of the latest scientific information available in the CAS Content Collection, offering a unique and unparalleled breadth of in-depth insights.

## Landscape Analysis of Parkinson’s Disease Research

### Journal Publication and Patent Trends

Our search in the CAS Content Collection^14^ for PD-related documents (search term: Parkinson* in Title, Abstract, Keywords, and Concept fields) retrieved over 250,000 scientific publications (journal articles and patents). For comparison, a similar number of publications are being retrieved from other popular science information databases such as Web of Science^18^ (∼220,000 retrieved documents) and PubMed^19^ (∼180,000 retrieved documents). Moreover, leveraging the strategic advantage of the CAS Content Collection, which provides information on most relevant substances and concepts identified by subject matter experts (SMEs) in the scientific publications, we identified the widely considered concepts within the PD-related publication subset. Additionally, we performed cross-concept searches to identify concept co-occurrence in the documents.

**Figure 1:**
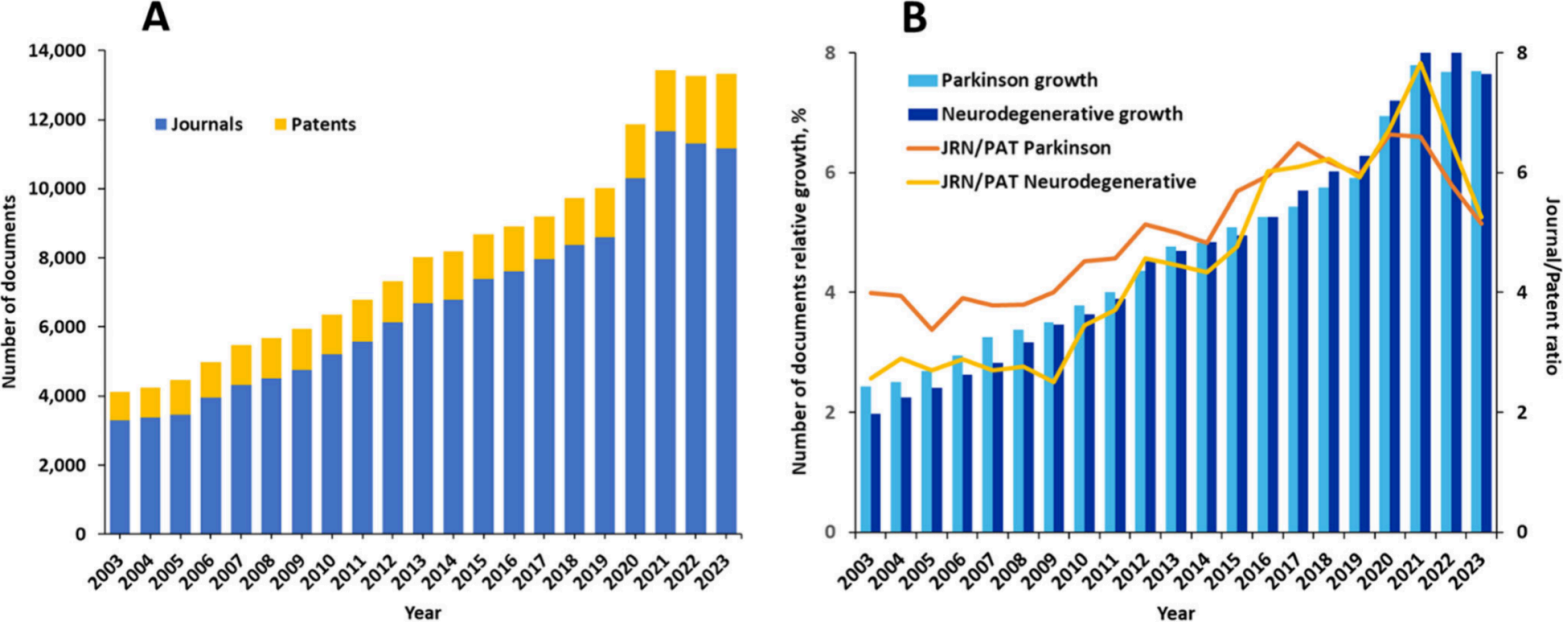
(A) Yearly growth of the document number (journal articles and patents) in the CAS Content Collection associated with PD. (B) Comparison between document growth in PD research (light blue bars) and overall neurodegenerative disease research (dark blue bars). Orange and yellow lines compare the journal/patent ratio for the class of PD and all neurodegenerative diseases, respectively.

There has been a steady growth of PD-related documents over the last two decades, with a >30% increase since 2019 (Figure 1A). The growth rate in PD research is similar to that in the general class of neurodegenerative diseases, with PD growth being faster in previous years (2023–2015) but slightly lagging recently (Figure 1B). The scientific journal publications are notably dominate (journal/patent ratio ∼4–6), but in the past three years the number of patents exhibited notable growth, associated with the initial accrual of scientific knowledge and its consequent transfer into patentable applications.

**Figure 2:**
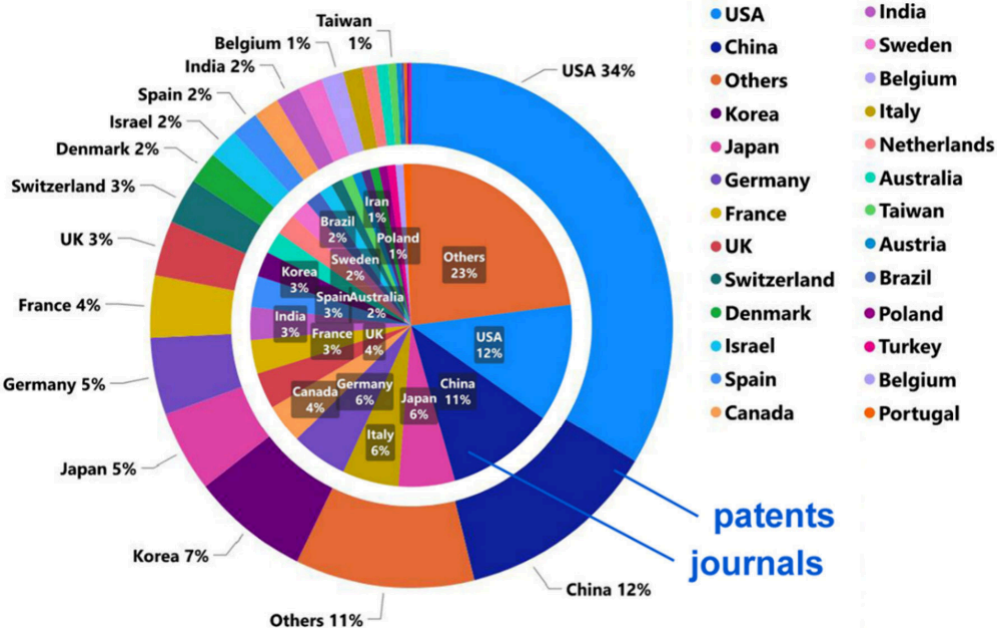
Leading countries by the percentage of PD-related journal articles (inner pie chart) and patents (outer donut chart). Source: CAS Content Collection.

**Figure 3:**
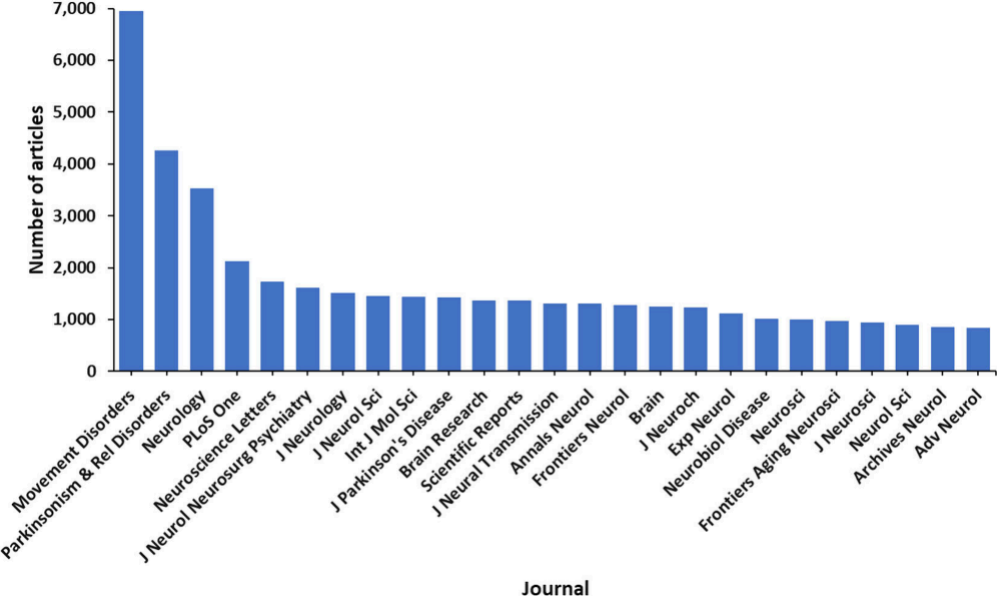
Top scientific journals in PD research based on publication data from the CAS Content Collection for 2003–2023.

The United States, China, Japan, South Korea, Germany, France, the United Kingdom, Italy, and Canada are the leading countries in terms of the number of published journal articles and patents related to PD research, with ∼1/3 of the patents originating from the United States (Figure 2). The journals *Movements Disorders*, *Parkinsonism & Related Disorders*, *Neurology*, and *PLoS One* have published the most articles related to PD research (Figure 3).

**Figure 4:**
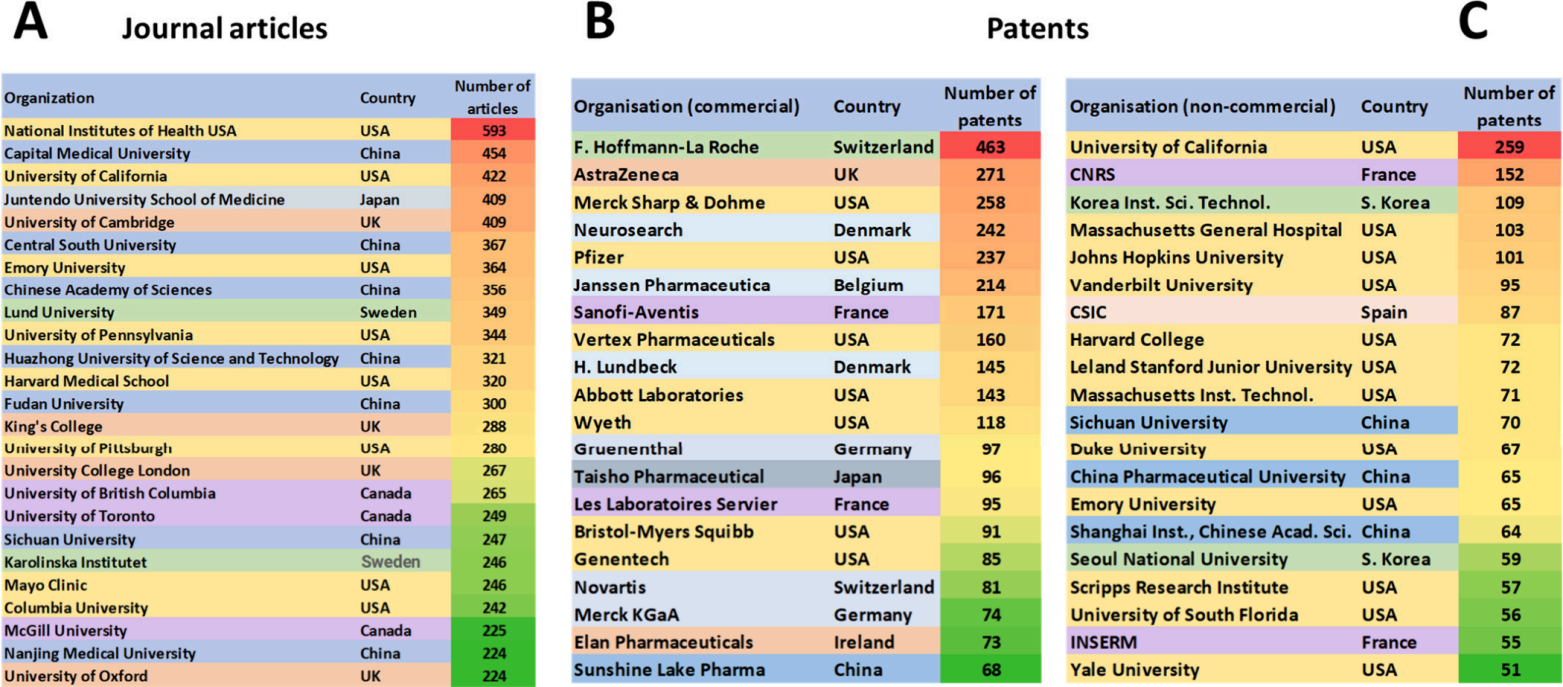
Top organizations in PD research with respect to published journal articles (A) and patents by commercial (B) and noncommercial (C) institutions.

The National Institutes of Health (NIH), USA, the Capital Medical University, China, the University of California, USA, Juntendo University School of Medicine, Japan, and the University of Cambridge, UK, have the most published articles in scientific journals (Figure 4A). Patenting pursuit is dominated by corporate companies as compared to academics (Figure 4B,C). F. Hoffmann-La Roche, AstraZeneca, Merck (MSD), Neurosearch, and Pfizer have the highest number of patent applications among commercial organizations (Figure 4B), while the University of California, CNRS (France), Korea Institute of Science and Technology, Massachusetts General Hospital, and Johns Hopkins University lead among the noncommercial organizations (Figure 4C).

**Figure 5:**
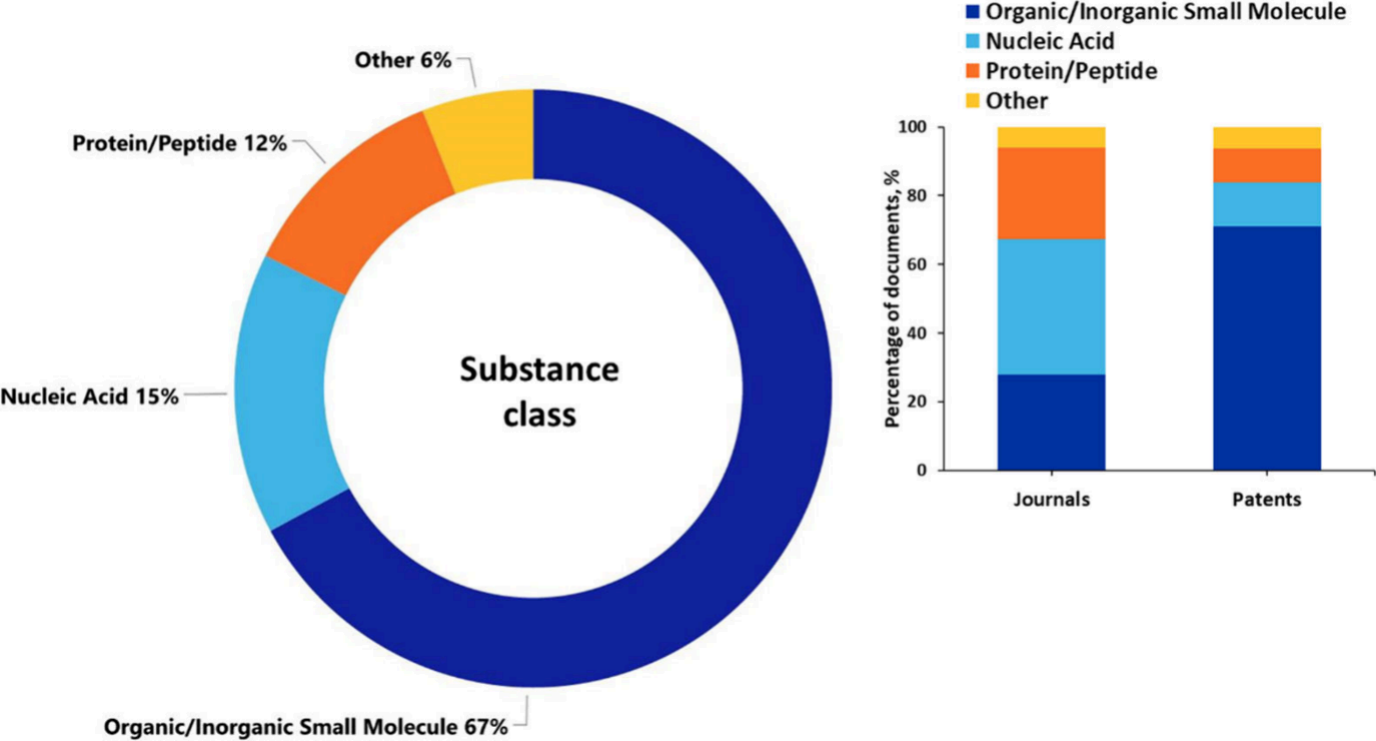
Distribution of the major substance classes in the documents related to PD. Inset: variation of the substance class distributions in journal articles and patents.

The class of organic/inorganic small molecules dominates the PD field (Figure 5). There is considerable variation in the distribution of substance classes between journal articles and patents. While small molecules strongly dominate in the patents (>70%), in the journal articles small molecules, nucleic acids, and proteins/peptides are equally represented (28%, 39%, and 27%, respectively) (Figure 5, inset). That difference is not unexpected since small molecule drugs are of certain commercial interest and thus more represented in patents, while journal articles discuss more fundamental features of the disease, thus discussing all three classes of substances.

## Etiology and Risk Factors of Parkinson’s Disease

The etiology of PD is complex and not fully understood. It is a multifactorial disorder affected by a combination of genetic, environmental, and age-related factors.^5−7^ While the exact cause of PD remains unclear, several risk factors have been identified that may increase an individual’s likelihood of developing the condition.^20−24^

Advancing age constitutes the primary risk factor for PD. The incidence of PD grows with age, with most cases diagnosed in individuals over the age of 60.^25^ As people age, there is a natural decline in the function and integrity of dopamine-producing neurons in the brain, which may contribute to the development of PD. However, PD can also affect younger adults, a condition known as early onset PD (EOPD).

While most cases of PD are sporadic (occurring randomly), around 10–25% are believed to have a genetic component.^26−29^ About 15–25% of people with PD have a family history.^1,29^ Individuals with a family history of PD are at a higher risk of developing it themselves. Having a first-degree relative, such as a parent or sibling, with PD increases the risk of developing the disease compared to individuals without a family history. Cases of EOPD are more likely to be familial than late-onset PD (LOPD), with about 20–30% of EOPD cases being familial compared to 10–15% of LOPD cases.^30^ Several genes have been implicated in familial forms of PD, including mutations in the SNCA (α-synuclein), LRRK2 (leucine-rich repeat kinase 2), Parkin (PARK2), PINK1 (PARK6), and DJ-1 (PARK7) genes.^31−34^ GBA (glucocerebrosidase) gene mutations are found in 8–14% of autopsy-proven PD diagnoses and increase the risk of developing PD.^29^ These genetic mutations can disrupt cellular processes involved in protein degradation, mitochondrial function, and oxidative stress response, leading to neuronal dysfunction and degeneration in the brain.

Exposure to specific environmental toxins and chemicals has been identified as a potential risk factor for developing PD.^35,36^ The most well-known environmental risk factor for PD is pesticide and herbicide exposure,^37^ especially those including compounds like rotenone and paraquat. Other likely environmental risk factors include industrial chemicals, heavy metals (e.g., lead, manganese), and certain solvents (e.g., trichloroethylene). Some studies have also suggested an association between PD risk and factors such as rural living, well water consumption, and exposure to certain metals in drinking water.^38^

Certain medical conditions and lifestyle factors have also been implicated as potential risk factors for PD, although their roles are less well-established.^39^ These include head injuries, prior exposure to viral infections, smoking, and caffeine consumption, and factors that contribute to these processes may influence disease risk.^40^

### Aging Is the Most Widely Explored Concept in Parkinson’s Disease Research

**Figure 6:**
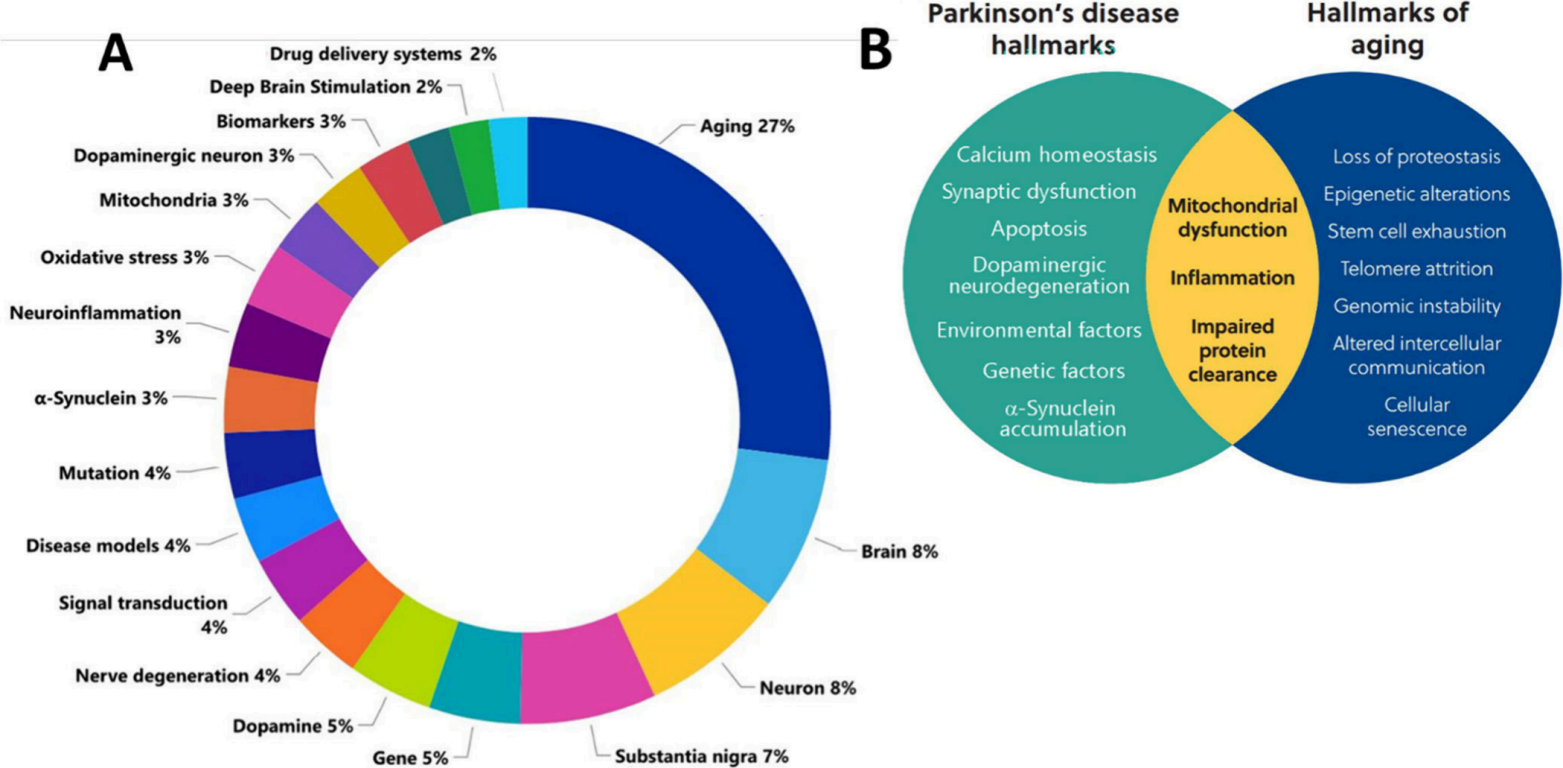
(A) Key concepts related to PD explored in the scientific publications. (B) Intersection between PD hallmarks and the hallmarks of aging. Source: CAS Content Collection.

We examined the assortment of PD-associated concepts in the published documents (journal articles and patents) (Figure 6A). Aging is the most widely explored concept; it has been discussed in nearly 1/3 of the PD-related published documents (Figure 6A). Aging is a leading risk factor for developing PD, as it is for the majority of neurodegenerative diseases, including Alzheimer’s disease, Huntington’s disease, and frontotemporal lobar dementia.^41,42^ While PD involves a complex range of symptoms, a key brain region affected by critical cell loss in PD is the substantia nigra, which is also the main reason for the motor symptoms associated with this disease. Specifically, it is the dopaminergic neurons of the pars compacta within the substantia nigra that are lost.^25^ The substantia nigra neurons are specific dopaminergic neurons in certain ways: they are pigmented (contain the pigment neuromelanin) and exhibit autonomous pacemaking activity and increased susceptibility to oxidative stress.^25,43−45^ They are also thought to be particularly susceptible to the mitochondrial dysfunction which accumulates within them with advancing age. Thus, understandably, the brain, neurons, and substantia nigra are also included in the top concepts considered in PD research (Figure 6A). These widely explored concepts in PD research typify the pathophysiological hallmarks of PD, which are discussed further in the text.

The development of PD and other neurodegenerative diseases is linked to the aging process.^46−50^ Specifically, mitochondrial dysfunction, inflammation, and impaired protein clearance are essential attributes of aging and PD (Figure 6B).

**Figure 7:**
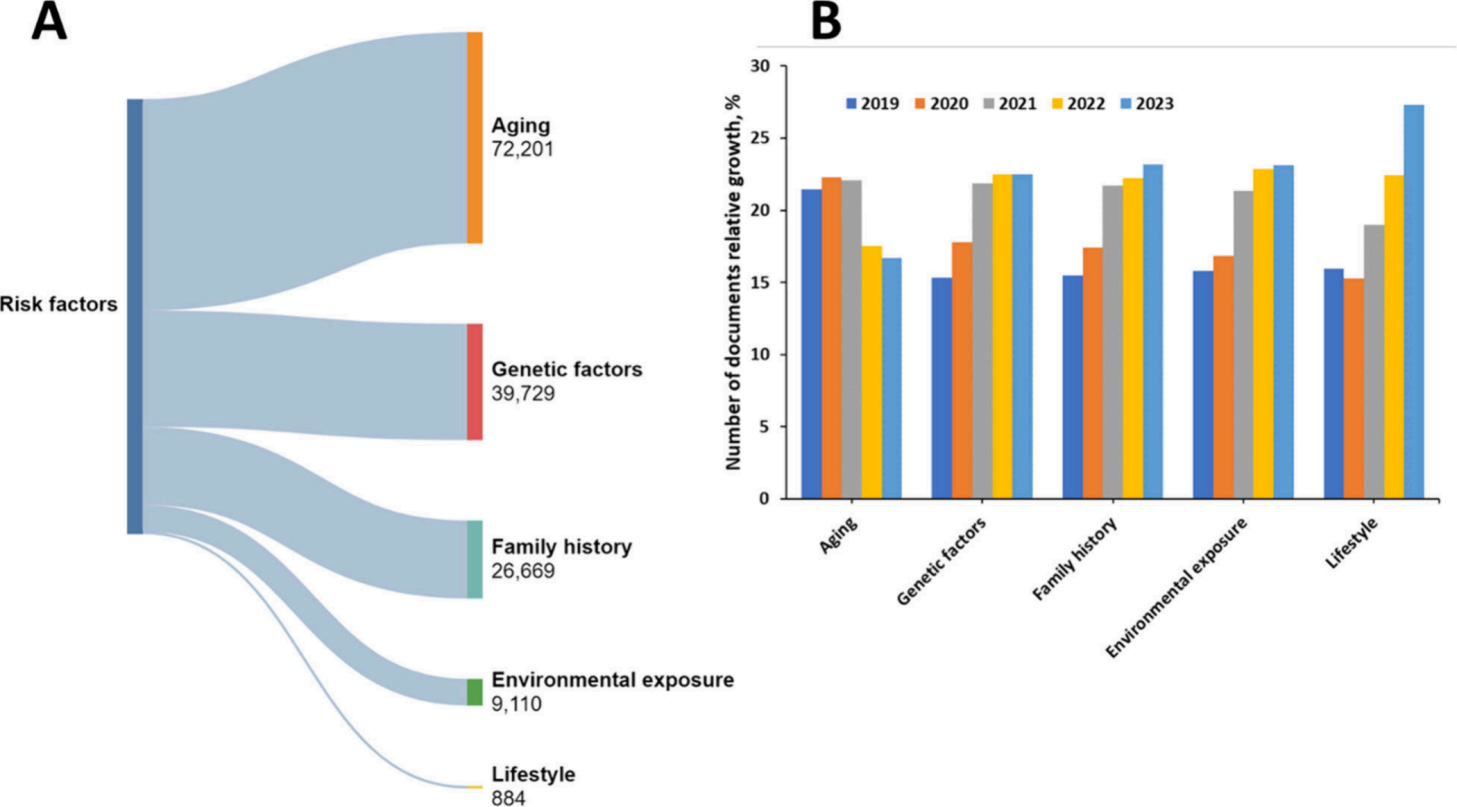
Risk factors for PD: (A) Allocation of documents associated with each risk factor within the CAS Content Collection. (B) Relative growth of the documents associated with the PD risk factors in the last 5-year period (2019–2023).

We further examined the CAS Content Collection documents associated with the PD research from the viewpoint of their relation to PD risk factors (Figure 7A) and their relative growth in the last five years (2019–2023) (Figure 7B). Understandably, the largest portion of the PD-related documents are associated with aging as a risk factor. A small number of documents are associated with lifestyle as a risk factor, but it has clearly attracted attention in recent years and exhibited the fastest growth.

### Pathophysiological Hallmarks of Parkinson’s Disease

The hallmarks of PD are the key pathological attributes and clinical manifestations that characterize the condition.^51−53^ These hallmarks encompass both the underlying molecular and cellular changes within the brain, such as dopaminergic neuron degeneration, α-synuclein pathology, and neuroinflammation, as well as the observable characteristic motor and nonmotor symptoms that define the clinical presentation of the disease. Understanding these hallmarks is crucial for elucidating disease mechanisms, developing diagnostic biomarkers, and identifying potential therapeutic targets for PD.

The hallmark pathological feature of PD is the progressive **degeneration of dopaminergic neurons** in the substantia nigra pars compacta, a brain region responsible for controlling movement.^51,54−57^ Dopamine is a neurotransmitter essential for movement control. This neuronal loss leads to a significant decline in dopamine in the basal ganglia, impairing motor control and contributing to the development of motor symptoms such as bradykinesia, tremor, rigidity, and postural instability.

Another characteristic attribute of PD is the buildup of abnormal protein aggregates termed Lewy bodies in neurons of PD patients.^58−62^ Lewy bodies, considered a pathological hallmark of PD, primarily consist of misfolded α-synuclein protein and are found in various brain regions, including the substantia nigra and other structures involved in motor and cognitive function. The presence of Lewy bodies is thought to contribute to neuronal dysfunction and degeneration, although the exact role they play in the pathogenesis of PD is still under investigation.

α-Synuclein aggregation and deposition are central to the underlying mechanisms of PD.^63−65^ α-Synuclein is a presynaptic protein involved in regulating neurotransmitter release. In PD, α-synuclein misfolds and aggregates into insoluble fibrils, forming Lewy bodies and Lewy neurites. This pathological accumulation of α-synuclein is believed to contribute to dopaminergic neuron dysfunction and degeneration.

Chronic inflammation and oxidative stress are believed to play significant roles in the progression of PD.^66−71^ Inflammatory processes in the brain, typified by microglial activation and increased pro-inflammatory cytokine production, can result in neuronal damage and contribute to the degenerative cascade, exacerbating disease progression. Inflammatory mechanisms may be triggered by α-synuclein pathology, oxidative stress, mitochondrial dysfunction, and other factors. Oxidative stress, which occurs when there is an imbalance between reactive oxygen species (ROS) and antioxidant defenses, can damage cellular components and exacerbate neuronal dysfunction and death.^40,72−75^

Motor symptoms are a hallmark clinical manifestation of PD and include bradykinesia, resting tremor, rigidity of muscles, and postural instability.^76−78^ These motor symptoms result from the progressive loss of dopaminergic neurons and the subsequent imbalance of neurotransmitters within the basal ganglia circuitry.

PD is associated with a wide variety of nonmotor symptoms that can significantly impact quality of life. These include cognitive impairment, psychiatric symptoms (such as depression, anxiety, and psychosis), autonomic dysfunction (e.g., constipation, urinary problems, orthostatic hypotension), sleep disturbances, sensory symptoms (e.g., hyposmia, visual disturbances), and others.^9,79,80^

Dysfunction of mitochondria has been implicated in the pathophysiology of PD.^81−86^ Mitochondrial impairment can lead to energy deficits, increased oxidative stress, and activation of cell death pathways, contributing to neuronal degeneration. Mutations in genes like PINK1 and Parkin, which are involved in mitochondrial function, have been associated with familial forms of PD.

Problems with protein clearance mechanisms, like autophagy and the ubiquitin-proteasome system, can lead to protein buildup and Lewy body formation. Impaired clearance of damaged or aggregated proteins can overwhelm cellular defenses and lead to neuronal toxicity and degeneration.^87−91^

### Genetic and Environmental Factors

While most cases of PD are sporadic, genetic factors contribute to disease susceptibility and may interact with environmental exposures.^26−28^ Mutations in genes such as SNCA (α-synuclein), LRRK2 (leucine-rich repeat kinase 2), Parkin, PINK1, and DJ-1 are associated with familial forms of PD, providing insights into disease mechanisms. Environmental factors, including exposure to various toxins, may elevate the risk of developing PD by promoting oxidative stress, mitochondrial dysfunction, and α-synuclein aggregation.^35−37^

**Figure 8:**
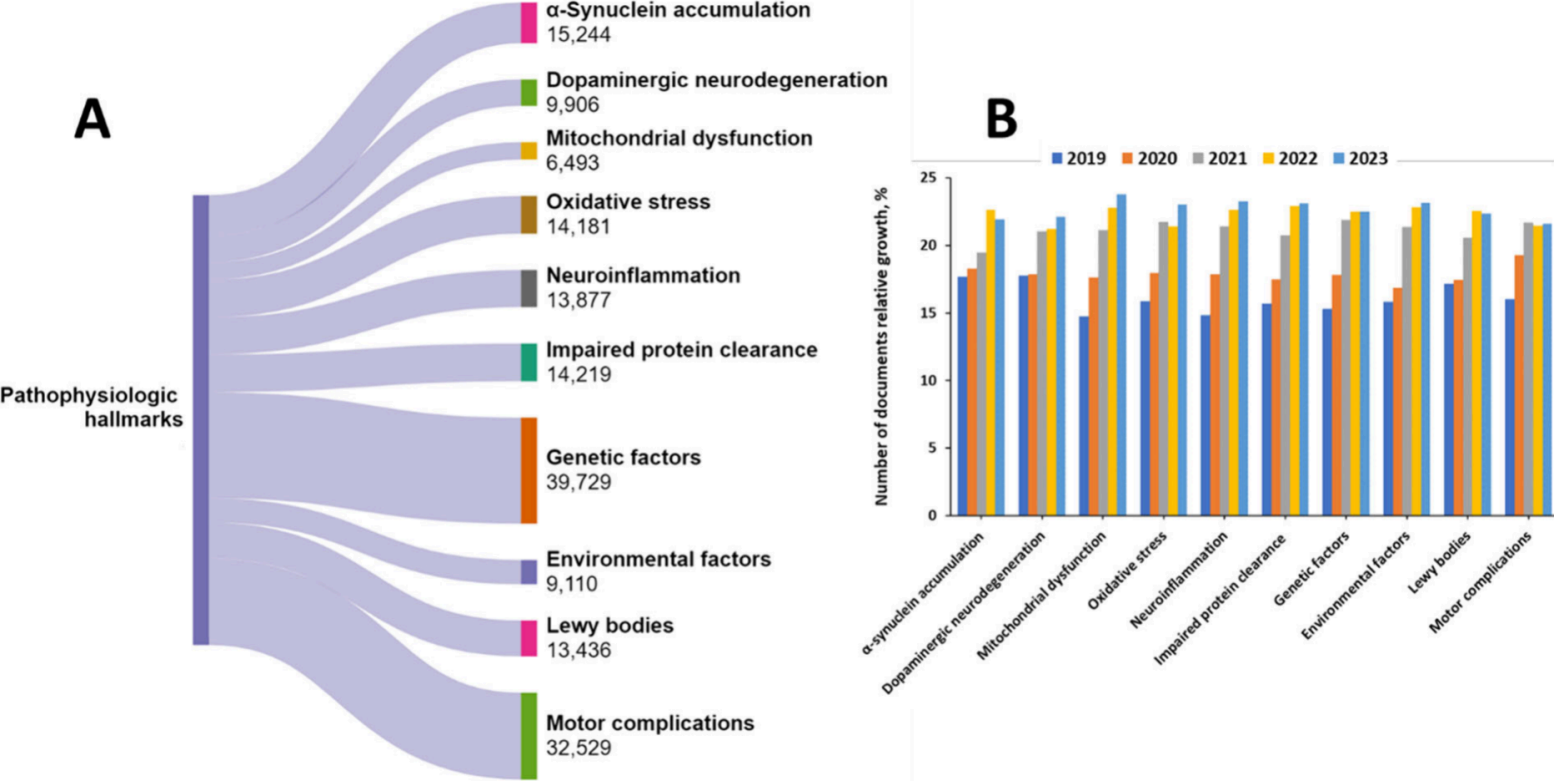
Pathophysiological hallmarks of PD: (A) Documents related to each hallmark within the CAS Content Collection. (B) Relative increase of the documents associated with the PD hallmarks in the last 5-year period (2019–2023).

We examined the documents in the CAS Content Collection associated with the PD research from the viewpoint of their relation to the pathophysiological PD hallmarks (Figure 8A), as well their relative growth in the last five years (2019–2023) (Figure 8B). The largest portion of the PD-related documents are associated with the genetic factors for PD pathophysiology, while the mitochondrial disfunction, along with neuroinflammation and environmental factors, exhibit the fastest and steady growth.

### Genetic Background of Parkinson’s Disease

The genetic background of PD encompasses a complex interplay of monogenic and polygenic risk factors, gene–environment interactions, and modifiers of disease penetrance and expressivity, which all contribute to disease susceptibility. While most PD cases are sporadic (without a known genetic cause), a small percentage of cases are considered familial, meaning they have a genetic component.^29,92−95^ Understanding the genetic basis of PD is essential for elucidating disease mechanisms, identifying novel therapeutic targets, and developing personalized treatment approaches for affected individuals.

Monogenic forms of PD are triggered by mutations in a single gene and are typically inherited in an autosomal dominant or recessive manner. Of the six genes linked to inherited PD, SNCA (PARK1 & 4) and LRRK2 (PARK8) cause autosomal-dominant forms, while Parkin (PARK2), PINK1 (PARK6), DJ-1 (PARK7), and ATP13A2 (PARK9) cause autosomal-recessive forms (Table 1).^13,29,94,96^

**Table 1:** Genes Linked to Heritable, Monogenic PD^a^

Gene	Symbol	Gene locus	Disorder	Inheritance
SNCA	PARK1	4q21–22	EOPD	autosomal-dominant
Parkin	PARK2	6q25.2–q27	EOPD	autosomal-recessive
SNCA	PARK4	4q21–q23	EOPD	autosomal-dominant
PINK1	PARK6	1p35–p36	EOPD	autosomal-recessive
DJ-1	PARK7	1p36	EOPD	autosomal-recessive
LRRK2	PARK8	12q12	Classical PD	autosomal-dominant
ATP13A2	PARK9	1p36	Kufor–Rakeb syndrome	autosomal-recessive

a EOPD, early onset PD.

- SNCA (PARK1–4) was the first gene linked to autosomal-dominant PD.^94^ Patients with such mutations typically develop EOPD (onset age ≤50). Mutations, duplications, or triplications in the SNCA gene lead to abnormal accumulation of α-synuclein protein, forming Lewy bodies, a pathological hallmark of PD.^97^ SNCA mutations are infrequent, and only a few specific mutations and gene duplications have been identified.^98^
- LRRK2 (PARK 8). Mutations in the LRRK2 gene are the most familiar source of late-onset familial PD, with a mutation frequency between 2% and 40% in different populations,^99−101^ and are associated with both autosomal dominant and recessive inheritance patterns.^94^ LRRK2-linked PD typically starts later in life and progresses slowly. LRRK2 mutations may increase the risk of developing sporadic PD as well.
- Parkin (PARK2) mutations are the most common cause of EOPD, being responsible for up to 77% of the familial cases with an onset age of <30 years and for 10–20% of all EOPD patients.^102,103^
- PINK1 (PARK6). Mutations in this gene are the second most common cause of autosomal recessive EOPD. PINK1 mutations occur in 1–9% of cases, varying across ethnic groups.^104−106^
- DJ-1 (PARK7) is the third gene linked to autosomal recessive PD, mutated in ∼1–2% of EOPD cases.^107^ Mutations in Parkin, PINK1, and DJ-1 genes are involved in mitochondrial function, oxidative stress response, and protein degradation pathways.^94^
- ATP13A2 (PARK9). Homozygous mutations in ATP13A2 cause an autosomal recessive atypical form of PD referred to as the Kufor–Rakeb syndrome, which exhibit juvenile onset, along with dementia, supranuclear gaze palsy, and pyramidal signs.^108^

### Polygenic Risk Factors

In addition to monogenic forms, PD risk is influenced by multiple genetic variants across the genome, each contributing small effects. Genome-wide association studies (GWAS) have identified numerous common genetic variants linked to PD risk. These variants are located in or near genes involved in various pathways, including α-synuclein aggregation, lysosomal function, immune response, and synaptic transmission.

Variants in several PD-related and a few other genes have been associated with an elevated PD risk. Specifically, GBA1 gene mutations are among the most common genetic risk factors for PD and related synucleinopathies.^109,110^ Over 495 diverse mutations have been identified in the GBA1 gene. These mutations can result in reduced enzyme activity, affecting lysosomal function and contributing to the accumulation of α-synuclein, a hallmark of PD.^111,112^

### Gene–Environment Interactions

While genetics play a significant role in PD risk, environmental factors also contribute to disease susceptibility, and gene–environment interactions may modulate risk. Environmental exposures, such as pesticides, herbicides, heavy metals, and traumatic brain injury, may interact with genetic factors to increase PD risk.

### Incomplete Penetrance and Variable Expressivity

Not all individuals with pathogenic mutations in PD-associated genes develop the disease, indicating incomplete penetrance. Variable expressivity refers to the wide range of clinical features and disease severity observed among individuals with the same genetic mutation.

**Figure 9:**
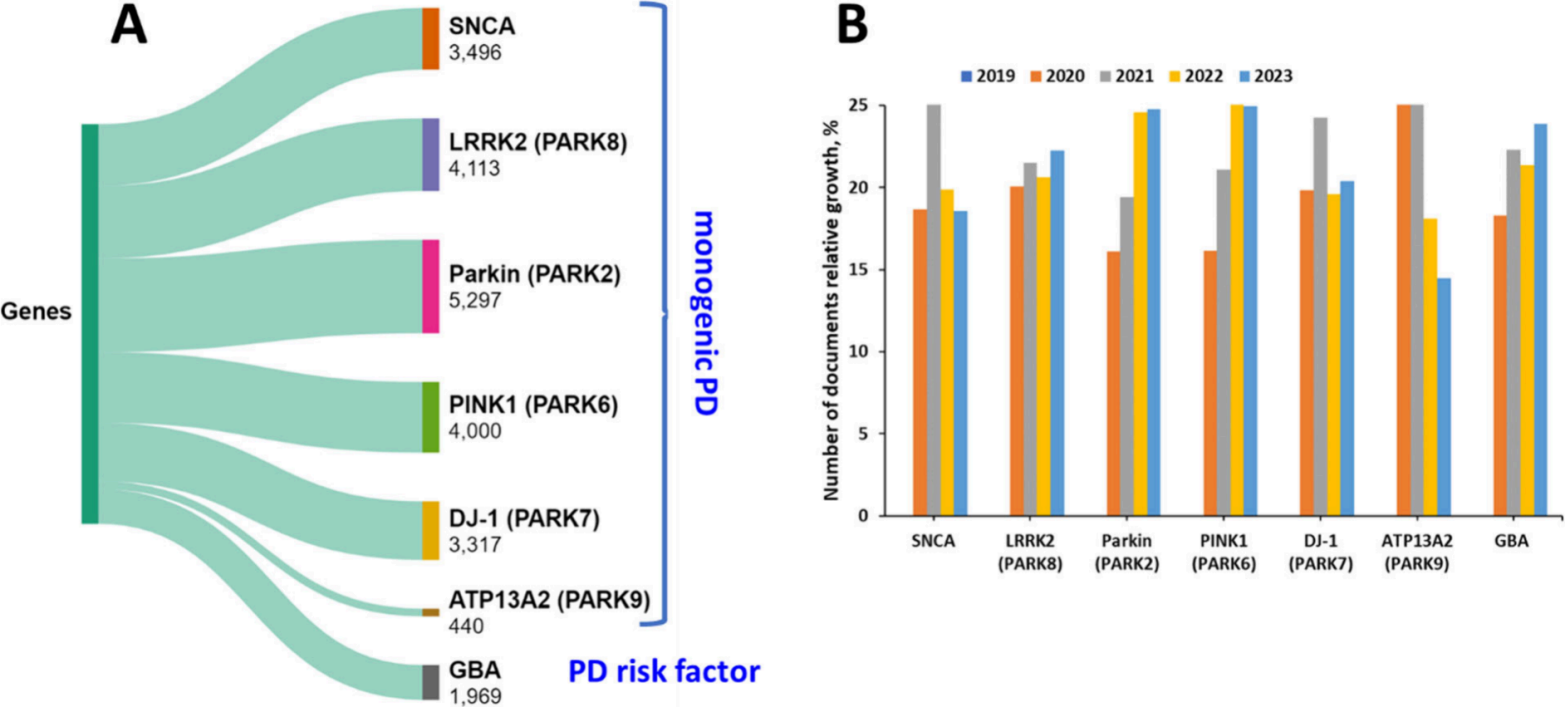
Key genes linked to familial PD: (A) Documents pertaining to these genes within the CAS Content Collection. (B) Relative growth of documents associated with these genes over the past 5 years (2019–2023).

We examined the documents in the CAS Content Collection associated with the PD research from the viewpoint of their relation to genes implicated in familial forms of PD (Figure 9A), as well their relative growth in the last five years (2019–2023) (Figure 9B). PARK2, PINK1, and GBA are those with the highest relative growth in recent years.

### Connection of Parkinson’s Disease with Other Diseases

PD is often accompanied by a wide range of comorbid diseases. Evidence shows that chronic diseases such as diabetes, depression, anemia, and cancer can be associated with the mechanism and advancement of PD.^113−119^ Studies have identified comorbid conditions such as bone fractures, cancer, dementia, diabetes, and stroke in patients with PD.^114,115,117^ Recent research indicates that certain comorbidities may enhance the risk of PD and precede the manifestation of motor symptoms. Furthermore, medications for treating diabetes and cancer have prompted neuroprotective effects in PD models. Yet, the mechanisms causing the co-occurrence of these diseases remain unclear.

**Figure 10:**
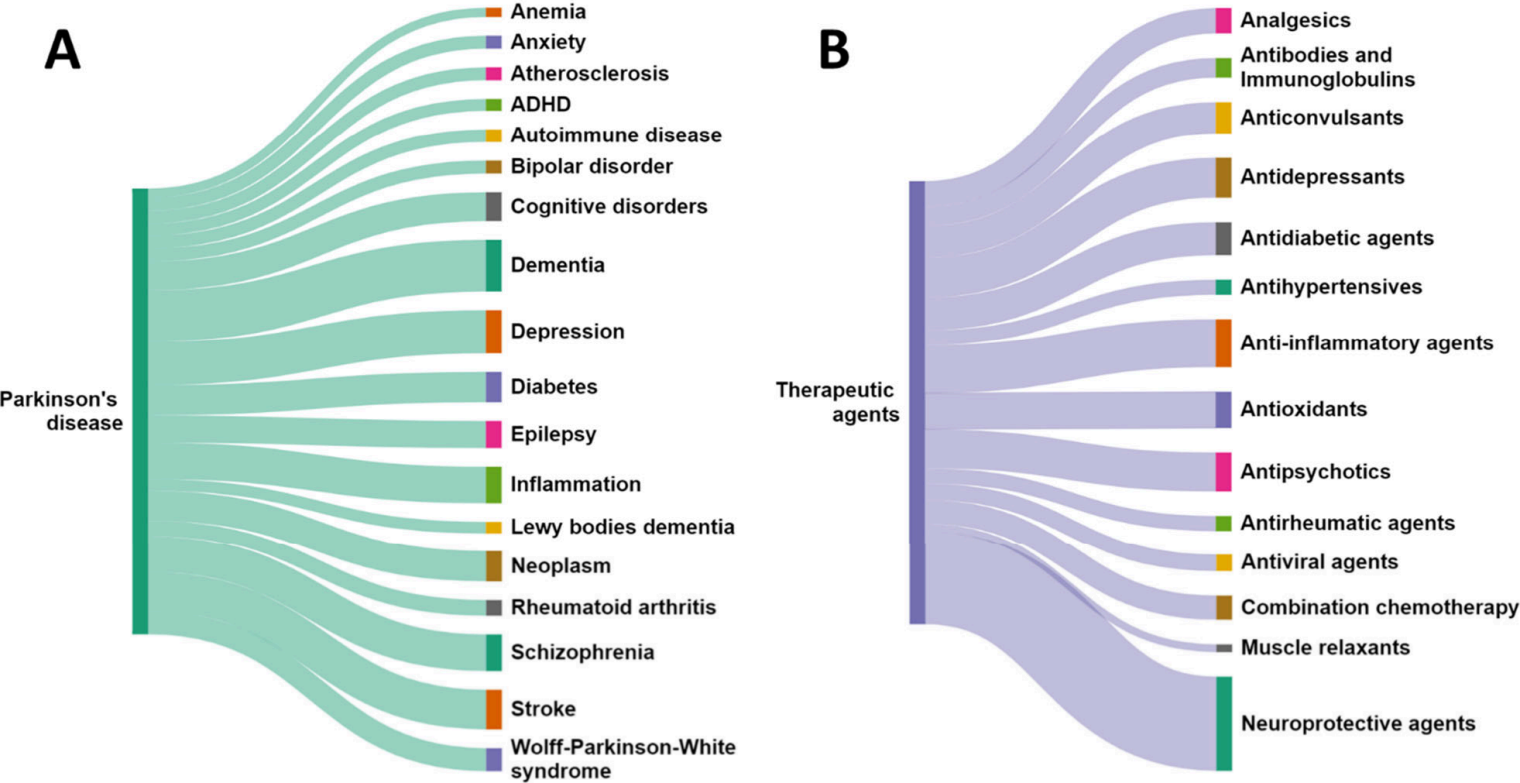
(A) Co-occurrence of PD and other disease concepts in the CAS Content Collection documents. (B) Therapeutic agent classes potentially examined for repurposing to PD therapy as estimated by their co-occurrence with the PD-related documents in the CAS Content Collection.

We analyzed the co-occurrence of diseases with PD as indicated by the co-occurrence of concepts in the documents included in the CAS Content Collection (Figure 10A). Dementia and inflammation are among the anticipated co-occurrences. Patients with PD frequently experience depression.^120^ Furthermore, depression has been suggested as a risk factor for PD. Inflammation has been commonly associated with depression and neurodegeneration. Proinflammatory cytokines trigger modifications in serotonin and dopamine neurotransmission, resulting in depression and PD.^121^ Yet the exact link between depression and PD remains unclear. The relationship between PD and diabetes was reported more than three decades ago^121^ and confirmed by multiple studies later on.^113^ PD and diabetes share comparable dysregulated routes such as inflammation, mitochondrial dysfunction, impaired autophagy, and insulin signaling,^122^ as well as certain genetic and environmental risk factors.^123−125^ Medications treating diabetes have indicated promise in alleviating motor symptoms in PD patients.^126^ Some studies link cancer and PD, indicating a decreased risk of PD among most of the cancer types: e.g., analysis has shown that diagnosis of PD is associated with a 27% decreased risk of cancer;^127^ another study showed a 17% reduced risk of cancer in PD patients.^128^ Prostate, lung, bladder, colorectal, blood, and uterus cancers have been reported as the most reduced in PD patients.^129^

### Diagnosis

The diagnosis of PD requires a comprehensive evaluation by a healthcare professional experienced in movement disorders, incorporating clinical judgment, symptom assessment, and supportive diagnostic tests as needed. Early diagnosis is crucial for initiating appropriate treatment and supportive care to improve outcomes and quality of life for patients with PD.^8,130,131^

Diagnosis of PD is primarily derived from clinical evaluation, including the assessment of motor symptoms, medical history, and neurological examination. While there is no definitive test to diagnose PD, healthcare professionals use established criteria and a combination of diagnostic tools to make an accurate diagnosis. A detailed physical examination is performed to evaluate motor function, including assessment of tremor, bradykinesia, muscle rigidity, and postural stability. Nonmotor symptoms, such as cognitive decline, psychiatric symptoms, autonomic dysfunction, and sleep disorders, may also be assessed during the evaluation. Response to dopaminergic medications, particularly levodopa (L-dopa), can provide supportive evidence for the diagnosis of PD.^132−134^

While neuroimaging is not typically required for diagnosing PD, it may be used to confirm the diagnosis and exclude other conditions that may mimic PD.^135−137^ Structural neuroimaging methods such as magnetic resonance imaging (MRI) may be used to exclude other causes of parkinsonism, such as tumors or stroke. Functional imaging techniques such as dopamine transporter (DAT) imaging with single-photon emission computed tomography (SPECT) or positron emission tomography (PET) can assess dopamine transporter activity in the brain, which is typically reduced in PD.

Several sets of clinical diagnostic criteria have been developed to aid in the diagnosis of PD, including the UK Brain Bank Criteria^138,139^ and the Movement Disorder Society (MDS) Clinical Diagnostic Criteria for PD.^140,141^ These criteria incorporate clinical features, response to medication, and exclusion of other parkinsonian syndromes to establish a diagnosis of PD.

### Biomarkers for Parkinson’s Disease

Biomarkers for PD are objective measures that can be used to detect, diagnose, and monitor disease progression; assess treatment response; and predict outcomes in affected individuals. Identifying reliable biomarkers is crucial for improving early diagnosis, developing disease-modifying therapies, and advancing personalized medicine approaches in PD.^142−153^ Overall, while several promising biomarkers for PD have been identified, further validation and standardization are needed to establish their clinical utility in diagnosis, prognosis, and therapeutic development. Integration of multiple biomarkers from different modalities may enhance diagnostic accuracy, improve patient stratification, and facilitate personalized treatment approaches in PD.

Genetic biomarkers (Table 2) have significantly advanced our understanding of PD. While most PD cases are infrequent, ∼10–25% are familial and can be attributed to specific genetic mutations. These genetic biomarkers can be classified into two categories: monogenic forms of PD (caused by mutations in a single gene) and genetic risk factors (variants that increase the risk of developing PD but do not directly instigate the disease). Pathogenic monogenic mutations in genes such as SNCA, LRRK2, and PARK2 are associated with familial forms of PD and may serve as biomarkers for genetic testing and personalized risk assessment. Genetic variants associated with PD risk identified through GWAS and next-generation sequencing may serve as genetic biomarkers for disease susceptibility and progression.^152,154−156^

**Table 2:** Genetic Biomarkers for PD

Genetic biomarker	Description	Clinical features
Monogenic Forms of PD		
SNCA^157,158^	SNCA gene encodes the alpha-synuclein protein, which is a major component of Lewy bodies, the pathological hallmark of PD. Mutations in SNCA (e.g., A53T, E46K) or multiplications of the gene (duplications or triplications) lead to increased alpha-synuclein aggregation.	Patients with SNCA mutations typically present with early onset PD, rapid disease progression, and more severe nonmotor symptoms including autonomic dysfunction and cognitive impairment.
LRRK2 (Leucine-Rich Repeat Kinase 2)^159,160^	Mutations in LRRK2 are the most well-known genetic cause of familial PD. The most prevalent mutation, G2019S, increases the kinase activity of LRRK2, leading to neuronal toxicity.	LRRK2-associated PD is clinically similar to idiopathic PD but can vary widely in age of onset and severity. The presence of LRRK2 mutations also increases the risk of PD in sporadic cases.
Parkin (PARK2)^161,162^	Parkin is an E3 ubiquitin ligase involved in mitochondrial quality control. Mutations in Parkin gene cause autosomal recessive juvenile parkinsonism (ARJP).	Parkin-related PD typically has an early onset (before age 40), with prominent dystonia and a slower progression compared to idiopathic PD. Patients often respond well to levodopa treatment.
PINK1 (PTEN-induced kinase 1)^163,164^	PINK1 is involved in mitochondrial quality control and works in conjunction with Parkin. Mutations in PINK1 lead to autosomal recessive EOPD.	PINK1-related PD presents with early onset, often before age 40, and includes motor symptoms like idiopathic PD. Patients may also show psychiatric symptoms like depression and anxiety.
DJ-1 (PARK7)^165−167^	DJ-1 is involved in oxidative stress response and mitochondrial function. Mutations in DJ-1 result in autosomal recessive EOPD.	Patients with DJ-1 mutations are present with early onset PD, usually before age 40, and have symptoms like idiopathic PD. Disease progression is typically slow.
Genetic Risk Factors		
GBA (glucocerebrosidase)^168,169^	Mutations in the GBA gene, which cause Gaucher disease when present in homozygous form, are significant risk factors for PD. Heterozygous GBA mutations impair lysosomal function and increase the risk of developing PD.	PD patients with GBA mutations are disposed to have an earlier onset, more severe motor symptoms, and a higher occurrence of cognitive impairment and psychiatric indications.
MAPT (microtubule-associated protein tau)^170,171^	Variants of the MAPT gene, which encode the tau protein, are associated with increased risk of PD. The H1 haplotype is particularly implicated in PD and other tauopathies.	MAPT variants are linked to cognitive decline and dementia in PD. However, their role in motor symptoms is less pronounced compared to other genetic factors.
LRRK2 (common variants)^159,172,173^	Beyond the pathogenic mutations, common variants in the LRRK2 gene, such as the G2385R and R1628P, are associated with an enhanced risk of PD, especially in certain populations.	These common variants are associated with a typical PD phenotype but with variability in age of onset and disease severity.
SNCA (common variants)^174,175^	Polymorphisms in the SNCA gene, such as the REP1 microsatellite repeat, are associated with enhanced risk of sporadic PD. These variants may affect alpha-synuclein expression levels.	Variants in SNCA are linked to earlier onset and increased severity of motor symptoms.
Genome-Wide Association Studies (GWAS) Identified Variants		
GWAS identified variants^176,177^	Some of the key loci identified include PARK16, BST1, HLA-DRA, RIT2, and GAK/DGKQ.	Variants at these loci confer a modest increase in PD risk and provide insights into the complex genetic architecture of PD. They highlight pathways that could be targeted for therapeutic intervention.

Proteomic biomarkers for PD (Table 3) provide valuable insights into the protein alterations associated with the disease. Proteomics, the large-scale study of proteins, enables the identification of disease-specific protein expression patterns, post-translational modifications, and protein interactions. These biomarkers can help with early diagnosis, following disease progression, and assessing treatment responses.

**Table 3:** Proteomic Biomarkers for PD

Proteomic biomarkers	Description	Biomarkers
α-Synuclein^178−181^	Presynaptic neuronal protein implicated in the pathogenesis of PD. Aggregation of misfolded α-synuclein into Lewy bodies is a hallmark of PD.	• Total α-synuclein: Reduced levels in cerebrospinal fluid (CSF) and blood plasma have been observed in PD patients.
		• Propagative α-synuclein seeds in serum: α-synuclein seeds associated with PD are present in the blood serum of patients with synucleinopathies.
		• Oligomeric and phosphorylated α-synuclein: Elevated levels of these modified forms in CSF and blood are associated with PD.
		• α-Synuclein in peripheral tissues: Detection of α-synuclein aggregates in skin biopsies and olfactory tissues can serve as peripheral biomarkers. Dermal phosphorylated α-synuclein has been identified by skin biopsy in 92.7% with PD.
DJ-1^165,166,182^	Involved in oxidative stress response and protection against mitochondrial dysfunction.	• Total DJ-1: Altered levels in CSF and blood have been reported in PD patients, with some studies showing reduced levels in CSF.
		• Oxidized DJ-1: Increased levels of oxidized DJ-1 in blood and CSF are associated with PD, reflecting oxidative stress.
Tau protein^183,184^	Microtubule-associated protein is involved in maintaining neuronal structure. Abnormal tau processing and aggregation are linked to neurodegeneration.	• Total tau: Elevated levels in CSF are found in PD patients, though not as high as in Alzheimer’s disease.
		• Phosphorylated tau (p-tau): Increased levels of p-tau in CSF can help differentiate PD from other neurodegenerative diseases.
Neurofilament light chain (NfL)^185−187^	The structural protein of neurons and its release into the CSF and blood is indicative of neuroaxonal damage.	• CSF and blood NfL: Elevated levels are observed in PD patients, particularly in those with more advanced disease and cognitive impairment.
Lysosomal proteins^188,189^	Lysosomal dysfunction is implicated in PD pathogenesis, particularly in cases with GBA mutations.	• Glucocerebrosidase (GCase): Reduced activity of GCase in CSF and blood is linked to PD, especially in GBA mutation carriers.
		• Cathepsin D: Altered levels in CSF have been associated with PD, reflecting lysosomal enzyme dysfunction.
Inflammatory and immune-related proteins^190,191^	Chronic neuroinflammation and immune activation are thought to contribute to PD progression.	• Cytokines and chemokines: Elevated levels of pro-inflammatory cytokines (e.g., IL-1β, IL-6, TNF-α) in blood and CSF are observed in patients with PD.
		• Complement proteins: Increased levels of complement components (e.g., C3, C4) in CSF indicate activation of the complement system in PD.
Oxidative stress markers^192,193^	Oxidative stress plays a crucial role in neuronal damage and PD pathogenesis.	• 8-Hydroxy-2′-deoxyguanosine (8-OHdG): Elevated levels in CSF and blood reflect oxidative DNA damage in PD.
		• Malondialdehyde (MDA): Increased levels of MDA, a marker of lipid peroxidation, are found in blood and CSF of PD patients.
Synaptic proteins^194^	Synaptic dysfunction and loss are key features of PD.	• Synaptosomal-associated protein 25 (SNAP-25): Reduced levels in CSF correlate with synaptic loss in PD.
		• Synaptotagmin-1: Decreased levels in CSF are associated with PD, reflecting synaptic vesicle dysregulation.
Mitochondrial proteins^81,195^	Mitochondrial dysfunction is a central feature of PD pathology.	• Cytochrome c: Increased levels in CSF and blood are indicative of mitochondrial damage and apoptosis.
		• Mitochondrial DNA (mtDNA): Elevated levels of cell-free mtDNA in blood and CSF reflect mitochondrial damage and have been associated with PD.
Other emerging proteomic biomarkers^196,197^	Growth Factors: Decreased amounts of brain-derived neurotrophic factor (BDNF) and glial cell line-derived neurotrophic factor (GDNF) in CSF are linked to PD.	• BDNF and GDNF in CSF.
	Metabolic Enzymes: Altered levels of enzymes involved in energy metabolism (e.g., creatine kinase) are observed in PD patients.	• Enzymes involved in energy metabolism (e.g., creatine kinase).

Metabolomic biomarkers (Table 4) are gaining traction in PD research due to their ability to provide comprehensive insights into the biochemical alterations associated with PD. Metabolomics enables the identification of disease-specific metabolic changes. Such biomarkers can help in early diagnosis, supervising disease progression, and evaluating treatment responses.

**Table 4:** Metabolomic Biomarkers

Metabolomic biomarkers	Description	Biomarkers
Energy Metabolism		
Glucose metabolism^198,199^	Impaired glucose metabolism has been implicated in PD, affecting neuronal energy supply and function.	Lactate: Increased levels in the CSF and blood of PD patients suggest altered glycolysis and mitochondrial dysfunction.
		Pyruvate: Elevated pyruvate levels indicate a shift in energy metabolism, possibly due to mitochondrial impairment.
Tricarboxylic acid (TCA) cycle^200,201^	TCA cycle is crucial for cellular energy production. Dysregulation of TCA cycle metabolites reflects mitochondrial dysfunction in PD.	Citrate and α-ketoglutarate: Altered levels in CSF and blood have been reported in PD patients, indicating disruptions in the TCA cycle.
Amino Acid Metabolism		
Branched-chain amino acids (BCAAs)^202,203^	BCAAs play a role in neurotransmitter synthesis and energy production. Dysregulated BCAA metabolism has been linked to neurodegeneration.	Valine, leucine, and isoleucine: Reduced levels in the blood and CSF of PD patients suggest altered BCAA metabolism.
Tyrosine metabolism^204,205^	Tyrosine is a precursor for dopamine synthesis, and its metabolism is crucial for maintaining dopaminergic function.	Homovanillic acid (HVA): Reduced levels of HVA, a dopamine metabolite, in CSF are indicative of dopaminergic neuron loss.
Lipid Metabolism		
Phospholipids^206,207^	Phospholipids are essential for cell membrane integrity and signaling. Altered phospholipid metabolism affects neuronal function and survival.	Phosphatidylcholine and phosphatidylethanolamine: Changes in the levels of these phospholipids in CSF and blood are associated with PD.
Sphingolipids^208,209^	Sphingolipids are involved in cell signaling and apoptosis. Dysregulated sphingolipid metabolism contributes to neurodegeneration.	Ceramides: Elevated levels in CSF and blood have been linked to PD, reflecting sphingolipid metabolism disturbances.
Oxidative Stress and Antioxidant Metabolism		
Oxidative stress markers^192,210^	Oxidative stress is a key factor in PD pathogenesis, leading to neuronal damage and death.	8-Hydroxy-2′-deoxyguanosine (8-OHdG): Increased levels in blood and CSF indicate oxidative DNA damage.
		Malondialdehyde (MDA): Elevated MDA levels, a marker of lipid peroxidation, are found in PD patients.
Antioxidant metabolism^73,193^	Antioxidants protect against oxidative stress. Alterations in antioxidant levels can indicate an imbalance in oxidative stress and defense mechanisms.	Glutathione: Reduced amounts in the brain and blood of patients with PD suggest impaired antioxidant defenses.
Neurotransmitter Metabolism		
Dopamine metabolism^211,212^	Dopamine deficiency is a hallmark of PD. Alterations in dopamine metabolites reflect dopaminergic neuron loss.	Dihydroxyphenylacetic acid (DOPAC) and homovanillic acid (HVA): Reduced levels in CSF and blood indicate impaired dopamine metabolism.
Serotonin and norepinephrine metabolism^213,214^	Serotonin and norepinephrine are also affected in PD, promoting nonmotor symptoms including depression and autonomic dysfunction.	5-Hydroxyindoleacetic acid (5-HIAA): Altered levels in CSF and blood suggest changes in serotonin metabolism.
Others		
Uric acid^215,216^	Uric acid is a natural antioxidant. Higher levels have been associated with a lower risk of PD, suggesting a protective effect.	Uric acid: Lower levels in blood and CSF are observed in PD patients, indicating reduced antioxidant capacity.
Microbial metabolites^217,218^	The gut microbiome is increasingly recognized for its role in PD pathogenesis. Gut bacteria-produced metabolites can influence brain function.	Short-chain fatty acids (SCFAs): Altered levels of SCFAs such as butyrate in feces and blood are associated with PD, reflecting changes in gut microbiome composition and activity.
Steroid metabolism^219,220^	Steroids, including neurosteroids, modulate brain function and inflammation. Altered steroid metabolism has been linked to PD.	Pregnenolone and DHEA: Changes in the levels of these neurosteroids in blood and CSF have been reported in PD patients.

Neuroimaging biomarkers (Table 5) play a crucial role in the diagnosis, monitoring, and understanding of PD. These biomarkers are derived from various imaging techniques that visualize structural, functional, and molecular changes in the brain.^136,150,221−223^

**Table 5:** Neuroimaging Biomarkers

Neuroimaging biomarkers	Description	Biomarkers
Structural Imaging		
Magnetic resonance imaging (MRI)^224,225^	Used to visualize brain anatomy and detect structural changes in PD.	• Substantia nigra (SN) changes: The dopaminergic neuron degeneration in the SN is a characteristic feature of PD. MRI can detect changes in the volume and iron content of the SN.
		• Diffusion tensor imaging (DTI), a type of MRI, measures the integrity of white matter tracts. Reduced fractional anisotropy in the SN and other brain regions indicates neurodegeneration.
		• Magnetization transfer imaging (MTI): MTI assesses changes in brain tissue microstructure. Decreased magnetization transfer ratios in the SN and other regions have been observed in PD patients.
Voxel-based morphometry (VBM)^226,227^	Analyzes MRI data to detect gray matter volume changes.	• Cortical and subcortical atrophy: PD patients often exhibit atrophy in cortical regions, such as the prefrontal cortex, and subcortical regions, such as the basal ganglia and thalamus.
Functional Imaging		
Positron emission tomography (PET)^228,229^	Used to assess brain metabolism, blood flow, and receptor binding.	• Dopaminergic function: PET tracers such as [18F]-DOPA, [11C]-raclopride, and [18F]-FP-CIT measure presynaptic dopamine synthesis, dopamine transporter density, and postsynaptic dopamine receptor availability. Reduced uptake in the striatum indicates dopaminergic neuron loss.
		• Glucose metabolism: [18F]-FDG PET measures regional brain glucose metabolism. Hypometabolism in frontal and parietal cortices and hypermetabolism in cerebellum and pons are distinctive of PD.
		• Neuroinflammation: PET tracers such as [11C]-PK11195 bind to the translocator protein (TSPO), a marker of microglial activation. Increased TSPO binding in the SN and other regions indicates neuroinflammation.
Single-photon emission computed tomography (SPECT)^230,231^	Assesses brain perfusion and dopamine transporter availability.	• Dopamine transporter imaging: SPECT tracers like [123I]-FP-CIT (DaTSCAN) bind to dopamine transporters. Reduced binding in the striatum helps differentiate PD from other parkinsonian syndromes.
		• Cerebral blood flow: SPECT perfusion imaging with tracers like [99 mTc]-HMPAO detects changes in regional cerebral blood flow. Hypoperfusion in the parietal and occipital cortices is often seen in PD.
Molecular Imaging		
PET with α-synuclein ligands^63,232^	α-Synuclein aggregation is a key feature of PD pathology. PET imaging with ligands targeting α-synuclein can visualize these aggregates.	• α-Synuclein aggregates: Ongoing research aims to develop PET ligands that specifically bind to α-synuclein aggregates, providing a direct measure of Lewy body pathology.
Iron Imaging		
Susceptibility-weighted imaging (SWI)^233^	Sensitive to magnetic susceptibility changes caused by iron deposition.	• Iron deposition: Increased iron content in the SN and other brain regions has been detected in patients with PD, associating with disease severity and progression.
Quantitative susceptibility mapping (QSM)^234,235^	Quantitatively measures magnetic susceptibility, providing detailed information on iron distribution.	• Iron accumulation: QSM reveals increased iron accumulation in the SN, basal ganglia, and other regions, serving as a marker of neurodegeneration.

Neurophysiological biomarkers (Table 6) are critical in understanding PD as they provide insights into the functional state of the nervous system. These biomarkers are derived from techniques that measure electrical activity and other physiological aspects of the nervous system.

**Table 6:** Neurophysiological Biomarkers

Neurophysiological biomarkers	Description	Biomarkers
Electroencephalography (EEG)		
Resting-state EEG^236,237^	Measures the brain’s spontaneous electrical activity at rest.	• Altered oscillatory activity: PD patients often exhibit enhanced power in the low-frequency bands (θ and δ) and reduced power in the high-frequency bands (α and β). This shift indicates changes in cortical excitability and network dynamics.
		• Event-related potentials (ERPs): Delayed and reduced amplitude of ERPs, such as the P300 component, have been observed in PD, reflecting cognitive impairments.
Task-related EEG^238,239^	Measures brain activity in response to specific tasks or stimuli.	• Movement-related potentials: Altered readiness potential and reduced amplitude of the Bereitschafts potential (BP) indicate impaired motor planning and execution in PD.
		• Cognitive task performance: Abnormal EEG patterns during cognitive tasks highlight deficits in attention, memory, and executive function in PD patients.
Magnetic encephalography (MEG)		
Magnetoencephalography (MEG)^240,241^	Measures the magnetic fields produced by neuronal activity, offering high temporal and spatial resolution.	• Altered cortical oscillations: Like EEG, MEG studies show increased low-frequency and decreased high-frequency oscillations in PD, particularly in motor and cognitive networks.
		• Functional connectivity: Reduced coherence and altered connectivity between cortical and subcortical regions reflect disruptions in network communication in PD.
Transcranial Magnetic Stimulation (TMS)		
Motor evoked potentials (MEPs)^242,243^	Measures the excitability of the motor cortex.	• Reduced MEP amplitude: Indicates impaired corticospinal excitability.
		• Prolonged central motor conduction time (CMCT): Reflects delayed neural transmission from the cortex to the spinal cord in PD patients.
Paired-pulse TMS^244,245^	Assesses intracortical inhibition and facilitation.	• Reduced short-interval intracortical inhibition (SICI): Indicates decreased GABAergic inhibition in the motor cortex.
		• Altered long-interval intracortical inhibition (LICI) and intracortical facilitation (ICF): Reflect changes in the balance between inhibitory and excitatory cortical circuits in PD.
Electromyography (EMG)		
Surface EMG^246−248^	Measures muscle electrical activity.	• Increased coactivation: Reflects increased simultaneous activation of agonist and antagonist muscles, contributing to rigidity in PD.
		• Tremor analysis: Characteristic 4–6 Hz resting tremor can be quantified to monitor disease progression and response to treatment.
Needle EMG^247,249^	Provides detailed analysis of muscle activity at the motor unit level.	• Motor unit potential changes: Alterations in motor unit recruitment and firing patterns indicate motor neuron dysfunction in PD.
Autonomic Function Tests		
Heart rate variability (HRV)^250,251^	Measures autonomic nervous system function.	• Reduced HRV: Indicates impaired autonomic regulation and is associated with nonmotor symptoms of PD such as orthostatic hypotension and cardiac dysautonomia.
Skin conductance response (SCR)^252,253^	Assesses sympathetic nervous system activity.	• Altered SCR: Reduced or absent skin conductance response reflects autonomic dysfunction in PD.
Others		
Event-related potentials (ERPs)^254,255^	Measures brain responses to specific sensory, cognitive, or motor events.	• P300 component: Reduced amplitude and prolonged latency of the P300 component are associated with cognitive impairments in PD.
		• N200 component: Altered N200 responses indicate deficits in conflict monitoring and executive function.
Quantitative sensory testing (QST)^256,257^	Assesses sensory perception thresholds.	• Altered pain and temperature thresholds: changes in thermal and pain perception thresholds suggest sensory dysfunction and altered pain processing in PD.
Polysomnography (PSG)^145,258^	Measures multiple physiological parameters during sleep.	• REM sleep behavior disorder (RBD): Increased muscle activity and abnormal behaviors during REM sleep are common in PD and can precede motor symptoms.
		• Sleep fragmentation and reduced sleep efficiency: Indicate sleep disturbances commonly seen in PD patients.

**Figure 11:**
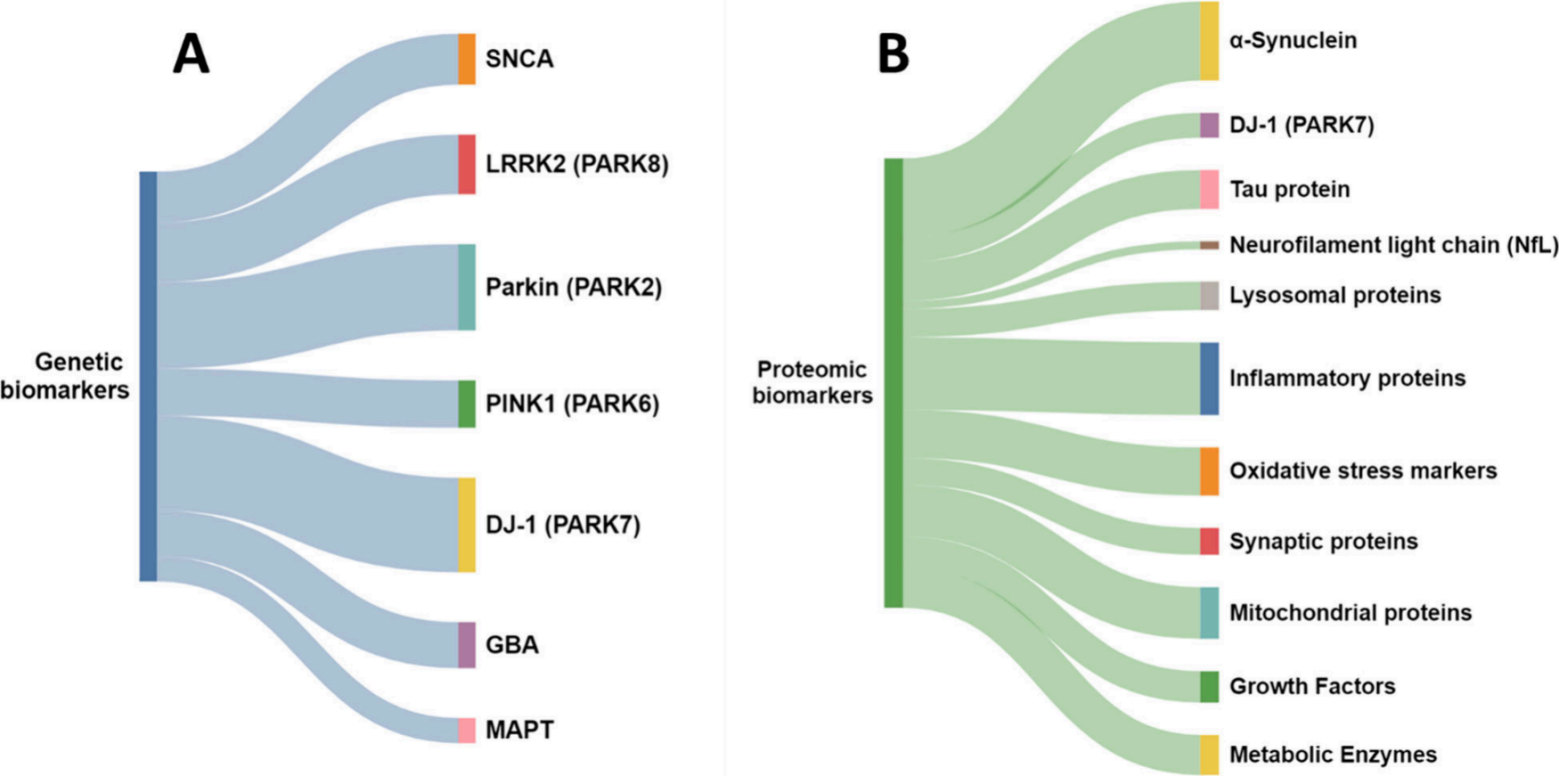
Distribution of documents related to major classes of (A) genetic biomarkers and (B) proteomic biomarkers within the CAS Content Collection.

The documents in the CAS Content Collection associated with the major classes of genetic and proteomic biomarkers are illustrated in Figure 11A and 11B, respectively.

While the advancements in PD biomarkers are promising, it is important to note that no single biomarker is yet definitive for diagnosing PD. Further research is needed to refine existing biomarkers and identify new ones. The goal is to develop a panel of biomarkers that can accurately predict the onset of PD, monitor disease progression, and guide treatment decisions.

### Treatment

Although there is presently no cure for PD, treatments exist that can help alleviate symptoms, enhance quality of life, and slow disease progression. Therapeutic approaches include pharmacotherapy with dopaminergic medications (e.g., levodopa, dopamine agonists, MAO-B inhibitors),^259^ deep brain stimulation (DBS) surgery,^260^ focused ultrasound,^261^ physical therapy, occupational therapy, speech therapy, and lifestyle modifications.^262,263^ Foslevodopa/foscarbidopa, marketed under the brand name Vyalev, a subcutaneous infusion of levodopa-based therapy, has been just approved by the FDA for treatment of motor fluctuations in adults with advanced PD.^264^ It is the first and only treatment for this condition. Another subcutaneous infusion of liquid levodopa/carbidopa, ND0612, tested as a potential treatment in PD, had supportive data from the pivotal phase 3 BouNDless trial (NCT04006210), a large-scale study of patients with PD.^265,266^

The specific treatment plan is tailored to everyone based on their symptoms, disease stage, and overall health. Additionally, ongoing research continues to explore potential therapies intended for slowing or halting the progression of the disease. Early diagnosis and intervention are crucial in managing PD and improving quality of life for affected individuals. Overall, the management of PD is multidisciplinary and requires a collaborative approach involving healthcare professionals, patients, and caregivers. By addressing motor and nonmotor symptoms, optimizing medication regimens, promoting healthy lifestyle habits, and providing supportive care, it is possible to enhance quality of life and functional independence for individuals living with PD.

## Clinical Trial Research

**Figure 12:**
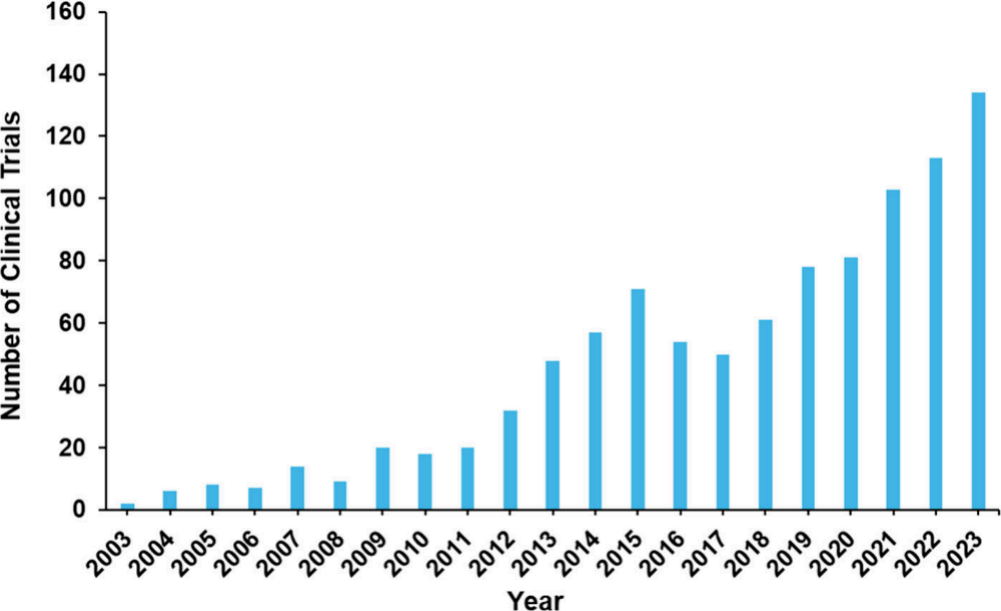
Number of PD therapeutic clinical trials by year.

Clinical trials currently exploring treatment of PD are surveyed in this section to understand the current stage of clinical development. Around 1000 clinical trials have been listed on clinicaltrials.gov in the recent 10 years aimed at the treatment of PD, showing continued growth of clinical development. Figure 12 shows a rising oscillating curve through 2018, with continued upward growth onward, starting at just a few clinical trials in 2003 and rising to around 150 clinical trials in 2023.

**Figure 13:**
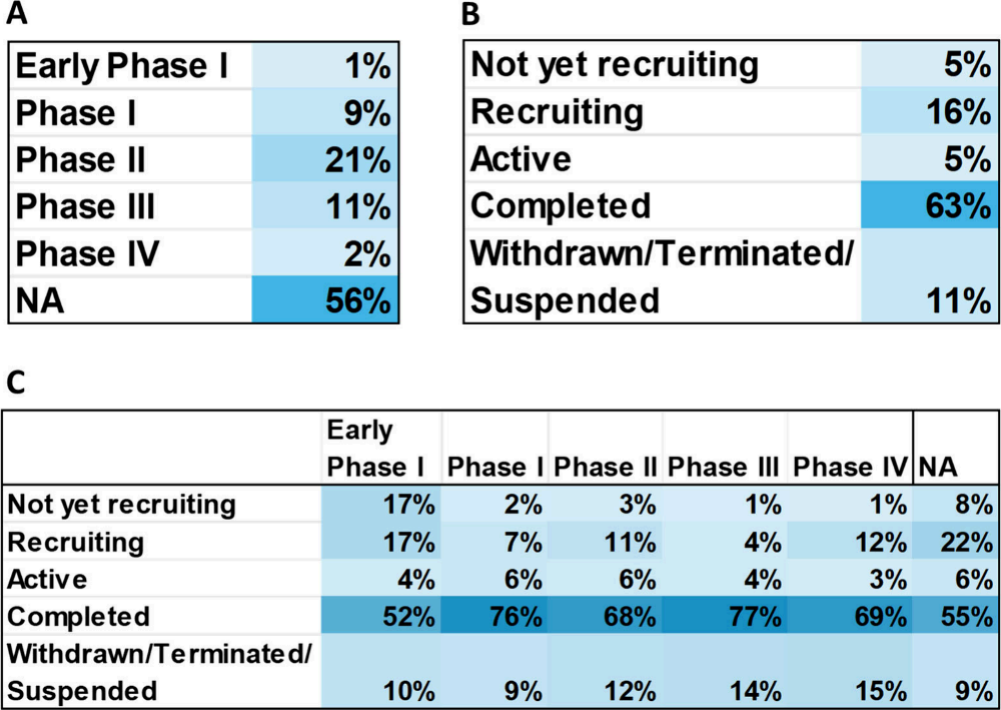
Percentage of PD clinical trials: (A) total clinical trial phase development; (B) total clinical trial statuses; and (C) clinical trial phases against statuses. Phase I/II and Phase II/III studies are classified under Phase II and Phase III studies, respectively.

Examination of PD clinical trials shows that ∼56% of all trials have not been phased, and these trials include interventions without FDA-defined phases such as medical devices or behavior interventions (Figure 13A). Phase II (21%) and Phase III (11%) studies are the next largest groups of trials. This indicates current efforts are being placed on researching newer anti-Parkinson’s agents and testing their safety and efficacy. Over 60% of all clinical trials in the recent 10 years have been completed (Figure 13B). The recruiting status (16%) is the next largest group of trials, which is encouraging, meaning that new clinical trials are created and executed. Early Phase I studies have the largest percentage of all phases for both the not yet recruiting (17%) and recruiting (17%) statuses, signaling that newer therapeutics are getting ready to enter the earliest stages of human studies by evaluating their safety and appropriate dosing regimens (Figure 13C).

### Current Treatment and Management of Parkinson’s Disease

PD currently has no cure, but there are several treatments available to manage its symptoms and improve quality of life. Managing PD often requires a comprehensive approach tailored to the individual’s symptoms and needs.^130,267−271^

## Medications

- **Levodopa (L-dopa)** is the most efficient medication for managing motor symptoms of PD.^272,273^ It is converted to dopamine in the brain, enhancing motor performance. Levodopa is combined with carbidopa or benserazide, which prevents the peripheral conversion of L-dopa to dopamine outside the brain, lowering peripheral side effects such as nausea. Common brand names include Sinemet and Madopar. Another preparation, Stalevo, contains levodopa and carbidopa and also the COMT inhibitor entacapone.^274^
- **Dopamine Agonists** mimic the action of dopamine in the brain. They are applied as monotherapy or in combination with levodopa.^275,276^ They stimulate dopamine receptors and help alleviate motor symptoms. Examples include pramipexole (Mirapex), ropinirole (Requip), rotigotine (Neupro), and apomorphine (Apokyn).
- **Monoamine Oxidase Type B (MAO-B) Inhibitors** block the enzyme monoamine oxidase type B, which metabolizes dopamine in the brain, thereby increasing dopamine levels.^277,278^ They can be used as monotherapy in early PD or as adjunctive therapy with levodopa. Examples include selegiline (Eldepryl, Zelapar) and rasagiline (Azilect).
- **Catechol-O-methyltransferase (COMT) Inhibitors** extend the effects of levodopa by inhibiting its breakdown, reducing motor fluctuations.^279,280^ They are often used in combination with levodopa/carbidopa. Examples include entacapone (Comtan), tolcapone (Tasmar), and opicapone (Ongentys).
- **Anticholinergic drugs** may be used to manage tremors and dystonia in some PD patients. They work by blocking the action of acetylcholine, a neurotransmitter involved in motor control. Examples include trihexyphenidyl (Artane) and benztropine (Cogentin).
- **Amantadine** can provide relief from dyskinesias (involuntary movements) and may also have modest benefits for other PD symptoms.^281,282^ Its specific mechanism of action in PD is not fully known but may involve dopamine release and NMDA receptor antagonism. Commonly used as an adjunctive therapy in advanced PD.
- **Adenosine A2A Receptor Antagonists** are newer drugs that enhance dopaminergic neurotransmission by blocking adenosine A2A receptors.^283−285^ They may help improve PD symptoms by reducing “off” time and improving motor function. These drugs may work similarly to increasing dopamine in the basal ganglia, which is involved in PD symptoms. Adenosine A2A receptors are located near dopamine receptors in the basal ganglia, and adenosine slows movement while dopamine controls it. Examples include Istradefylline.

Currently, levodopa is considered the most successful medication for PD symptoms. Previous studies have suggested that in certain instances other medications such as MAO-B inhibitors, amantadine, anticholinergic agents, β-blockers, or dopamine agonists can be introduced initially to avoid levodopa-related motor complications. However, long-term follow up to the levodopa in early PD (LEAP) study has found no difference in prevalence of motor complications between early and late introduction of levodopa therapy.^286^ Recent meta-analysis concluded that delaying treatment with levodopa does not appear to prevent levodopa-related motor complications in PD. Indeed, modifying the levodopa dosing schedule or combining it with other medications, such as MAO-B inhibitors, catechol-O-methyltransferase inhibitors, or dopamine agonists, may help manage motor fluctuations. Reducing or withdrawing dopaminergic medication, particularly dopamine agonists, may help manage impulse control disorders.^287^ Thus, additional treatment with dopamine agonists may reduce the requirement for higher doses of levodopa and therefore reduce the risk for dyskinesias; however, such practice is often associated with a higher frequency of adverse effects related to dopamine agonists.^288^ According to recent updates from the Movement Disorder Society, levodopa remains the recommended first-line treatment for PD as it is considered the most efficient medication for managing motor symptoms like tremor, rigidity, and bradykinesia, despite potential side effects that may arise with long-term use.^130,289^ Along these lines, making use of levodopa and dopamine agonists for treating motor symptoms at all stages of PD has been proven beneficial. Dopamine agonists and drugs blocking dopamine metabolism are efficient for treating motor fluctuations. Regarding nonmotor symptoms, clozapine has been found effective for hallucinations; cholinesterase inhibitors may improve symptoms of dementia; and antidepressants and pramipexole may improve depression.^287^

Table 7 summarizes common PD medications for treating motor symptoms, with their chemical structures, indications, and an exemplary clinical trial.^287^

**Table 7:**
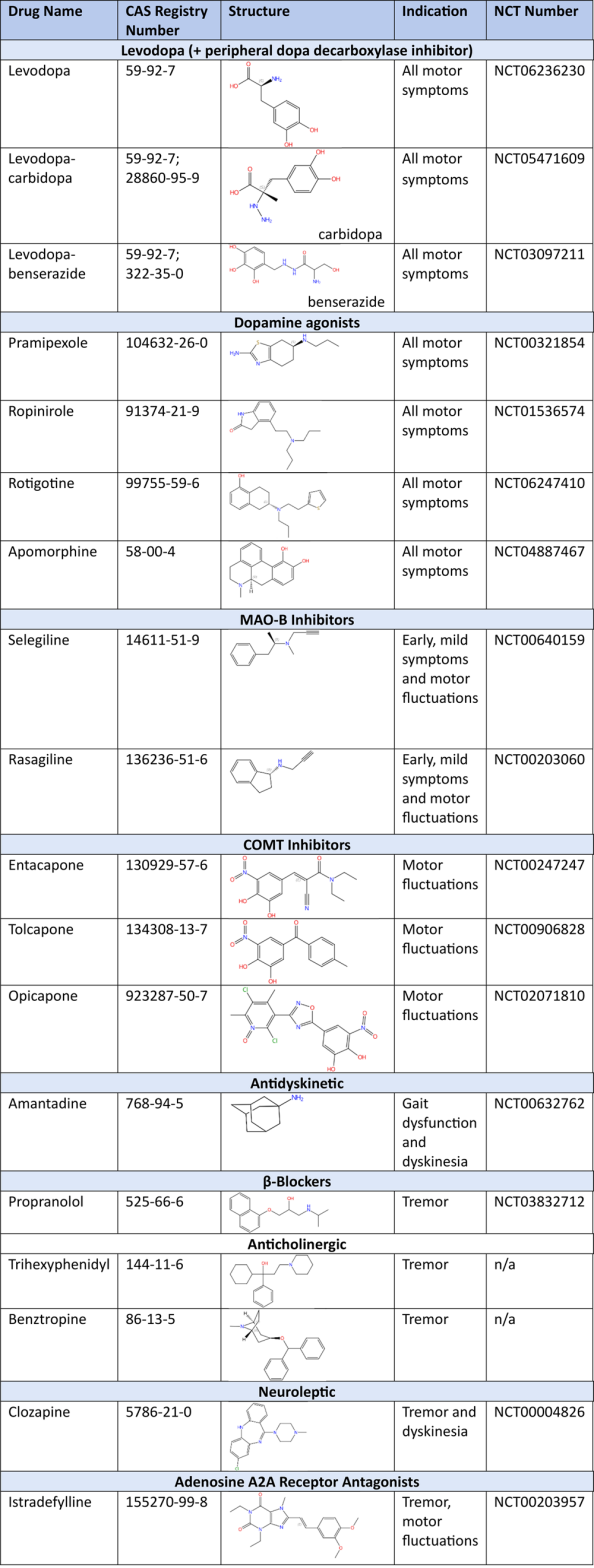
Treatment of Motor Symptoms of Parkinson’s Disease

Along with motor symptoms associated with PD, nonmotor symptoms such as cognitive issues, mood disorders, sleep disturbances, and others can significantly impact a person’s quality of life.^290−292^Table 8 lists some exemplary common nonmotor symptom treatments.^293,294^

**Table 8:** Treatment of Nonmotor Symptoms of PD

Drug class	Mechanism of action	Examples
Antidepressants^287,295^	Several classes, such as selective serotonin reuptake inhibitors (SSRIs) and tricyclic antidepressants (TCAs), are used to treat depression in PD.	SSRIs (sertraline, citalopram), TCAs (amitriptyline)
Anxiolytics and sleep aids^294,296^	Address anxiety and sleep disturbances commonly seen in PD.	Melatonin
Cognitive enhancers^297−299^	Cholinesterase inhibitors increase acetylcholine levels in the brain to help manage cognitive impairment.	Rivastigmine, donepezil
	NMDA receptor antagonists modulate glutamatergic neurotransmission, which can be beneficial in neurodegenerative diseases.	Memantine

### Drug Repurposing

Given the absence of a cure for PD, drug repurposing studies have been eagerly looking to identify current drugs to be repurposed to treat PD. In view of the time-consuming and costly nature of the pharmaceutical development process, drug repurposing offers a promising avenue to accelerate the development of PD treatments. By exploring the PD-related effects of agents approved for other disorders, researchers can leverage their established safety profiles, pharmacokinetic descriptions, formulations, dosages, and manufacturing procedures. For example, repurposing of glucagon-like peptide-1 (GLP-1) receptor agonists, commonly used as type 2 diabetes medication, for treating PD is rationalized by several promising findings. In some studies, the prevalence of PD has been reported to be lower in patients with diabetes who have been treated with GLP-1 receptor agonists or dipeptidyl peptidase-4 inhibitors, which enhance GLP-1 levels, than in patients who received other diabetes medications.^300,301^ Indeed, both type 2 diabetes and PD share similar pathological mechanisms, including insulin resistance, mitochondrial dysfunction, oxidative stress, and chronic inflammation. GLP-1 receptor agonists, used in type 2 diabetes, can address these mechanisms, potentially benefiting PD patients.^302,303^*In silico* pharmacology and AI are being used to identify potential drug repurposing candidates for PD. The occurrence of certain drug class concepts within the PD research area within the documents of the CAS Content Collection are illustrated in Figure 10B. Exemplary drugs for PD repurposing are summarized in Table 9.^303−334^

**Table 9:**
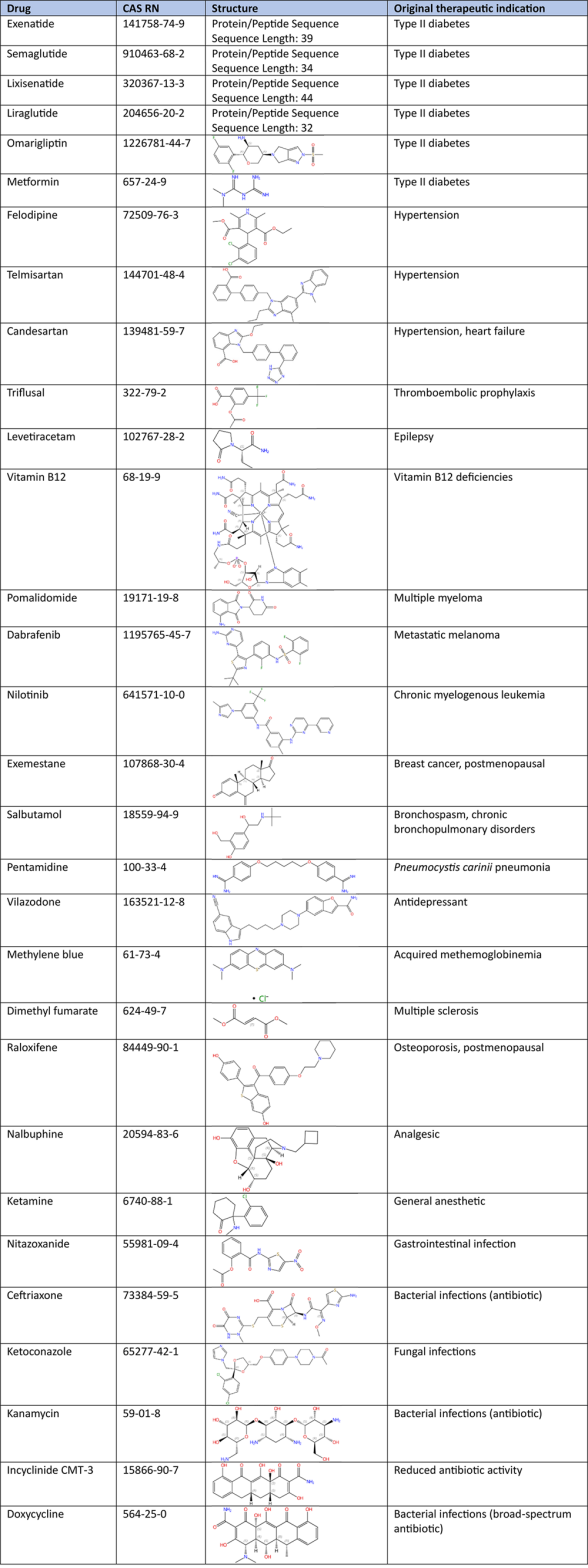
Exemplary Drugs Commonly Considered for Repurposing to PD

## Surgical Interventions

Deep brain stimulation implants electrodes into specific brain regions, usually the subthalamic nucleus (STN) or the globus pallidus internus (GPi), and delivers continuous, high-frequency electrical impulses to modulate abnormal neuronal activity.^260,335^ This modulation can help normalize abnormal brain activity associated with PD, improve motor symptoms, and reduce medication-related complications in certain patients. Another intervention, Duopa pump therapy, includes a gel formulation of levodopa and carbidopa (Duopa) that is delivered continuously through a pump system directly into the small intestine. It can provide more stable levodopa levels and improve motor fluctuations in advanced PD.^336−338^ Exablate neuro, a focused ultrasound treatment, is used to target and ablate the globus pallidus to treat mobility symptoms, tremor, rigidity, and dyskinesia in both tremor-dominant and advanced PD patients.^261^

## Rehabilitative Therapies

Physical therapy can help improve mobility, balance, flexibility, and strength in PD patients. It may also include exercises to address gait abnormalities and freezing of gait.^339−341^ Occupational therapy can help PD patients develop strategies to perform daily activities more independently and safely and recommend assistive devices and modifications to the home environment.^263,342^ Speech therapy can help PD patients to address speech and swallowing difficulties, improve vocal projection, and teach techniques to enhance communication.^263,343^

## Lifestyle Modifications

Regular exercise, including aerobic activities, strength training, and balance exercises, can help improve motor function, mobility, and overall well-being in PD patients. Moreover, it has been recently suggested that incorporating high-intensity exercise into daily routine can be a powerful tool in managing PD.^344−,346^ A healthy diet may support overall health and help manage constipation, a common nonmotor symptom of PD. Stress management techniques such as mindfulness, relaxation exercises, and stress reduction strategies may help alleviate anxiety and improve coping mechanisms.^347,348^

### Drug Delivery Systems

While there is currently no cure for PD, there are medications that can help to manage the symptoms. However, these medications can have limitations, such as short-lived effects and difficulty crossing the blood–brain barrier (BBB). The BBB is a highly selective barrier that protects the brain from harmful substances in the bloodstream. Unfortunately, it also prevents many drugs from reaching the brain, where they are needed to treat PD. Drug delivery systems are being developed to overcome these limitations. Drug delivery systems are designed to deliver drugs to particular sites in the body, including the brain. For PD, drug delivery systems are being investigated to (i) improve the delivery of drugs to the brain; (ii) reduce harmful side effects; and (iii) provide sustained release of medication.

Pharmaceutical nanotechnologies enable novel approaches to drug delivery.^349,350^ Nanoparticles, as drug carriers, impart advantages concerning improved efficacy and reduced adverse drug reactions. Pharmaceutical nanoparticles can overcome drug limitations like poor solubility and bioavailability. The major challenges in elaborating PD drug formulations include crossing through the blood–brain barrier and controlled drug release to prevent concentration fluctuations.

Pharmaceutical nanoparticles can be prepared from various materials offering variable physicochemical characteristics. Some of the most successful polymeric materials used include gelatin, alginate, hyaluronic acid, chitosan, poly(lactic-*co*-glycolic acid) (PLGA), polylactide, polyethylene glycol (PEG), and polycaprolactone.^351^ For instance, polymeric nanoparticles have been developed based on PEG–polycaprolactone, encapsulating Ginkgolide B, which is suggested to act as a neuroprotectant and treat PD.^352^ PLGA nanoparticles loaded with L-DOPA have been reported to increase motor function in PD patients.^353^ PLGA–PEG nanoparticles were engineered as carriers of coumarin, an effective inhibitor of monoamine oxidase B, which are able to cross intestinal and brain membranes, allowing the efficient transport of coumarin to the brain.^354^

Lipid nanoparticles are attractive nanocarriers for PD medications.^349,355^ Liposomes loaded with dopamine hydrochloride and functionalized with transferrin exhibited outstanding stability and improved ability to cross the blood–brain barrier.^356^ Semaglutide loaded liposomes demonstrated high stability, bioavailability, and passage through the blood–brain barrier and avoid toxic accumulation due to a half-life of approximately 1 week.^357^ Nalbuphine loaded solid–lipid nanoparticles allowed oral/nasal administration and greater dosage control.^358^

## Current Research and Future Directions on Parkinson’s Disease

Research into PD is an active and evolving field, with ongoing efforts focused on understanding disease mechanisms, identifying biomarkers, developing new treatments, and improving patient care. Ongoing research efforts in PD aim to deepen our understanding of disease mechanisms, advance precision medicine approaches, and develop new therapeutic strategies to improve outcomes for individuals living with PD. Collaboration among researchers, clinicians, patients, advocacy organizations, and industry partners is essential to accelerate progress and translate scientific discoveries into meaningful benefits for patients and families affected by PD.

### Disease Mechanisms and Pathogenesis

(i) Investigating the underlying mechanisms of PD pathology, including protein aggregation, mitochondrial dysfunction, neuroinflammation, and oxidative stress; (ii) Studying the role of genetic factors, environmental exposures, and epigenetic modifications in disease susceptibility and progression; (iii) Exploring the interplay between different cell types in the brain, including neurons, glial cells, and immune cells, in the development and progression of PD.

### Biomarkers for Early Detection and Diagnosis

(i) Identifying reliable biomarkers, such as imaging markers, fluid biomarkers (e.g., cerebrospinal fluid proteins), and genetic markers, for early detection and accurate diagnosis of PD; (ii) Developing noninvasive and accessible biomarkers that can be used in clinical practice to aid in disease monitoring, prognosis, and treatment response assessment.

### Neuroprotective and Disease-Modifying Therapies

(i) Developing neuroprotective and disease-modifying therapies intended for slowing or ceasing the progression of PD; (ii) Investigating novel pharmacological agents, gene therapies, and biologics targeting specific pathways implicated in PD pathology, such as α-synuclein aggregation, mitochondrial dysfunction, and neuroinflammation; (iii) Repurposing existing drugs or compounds with potential neuroprotective effects for PD treatment.

### Precision Medicine and Personalized Therapies

(i) Advancing precision medicine strategies to tailor treatments based on individual patient characteristics, including genetic profiles, biomarker profiles, and clinical phenotypes; (ii) Using omics technologies, including genomics, transcriptomics, proteomics, and metabolomics, to identify patient subtypes and predict treatment response.

### Nonmotor Symptoms and Quality of Life

(i) Investigating the underlying mechanisms of nonmotor symptoms in PD, such as cognitive impairment, psychiatric symptoms, autonomic dysfunction, and sleep disturbances; (ii) Developing targeted interventions and supportive care strategies to address nonmotor symptoms and improve quality of life for PD patients and their caregivers.

### Advanced Therapies and Surgical Interventions

(i) Advancing surgical interventions, including deep brain stimulation (DBS), focused ultrasound, and gene therapy, for the management of motor complications and nonmotor symptoms in PD; (ii) Exploring novel targets and techniques for neuromodulation and neurostimulation to improve treatment outcomes and reduce adverse effects.

### Digital Health and Technology Innovations

(i) Harnessing digital health technologies, including wearable devices, smartphone applications, and remote monitoring systems, for continuous monitoring of PD symptoms, motor fluctuations, and medication adherence; (ii) Integrating artificial intelligence (AI) and machine learning algorithms to analyze large-scale data sets and identify patterns in disease progression, treatment response, and patient outcomes.

### Clinical Trials and Translational Research

(i) Conducting meticulously designed clinical trials to assess the safety and efficacy of new therapies, including disease-modifying treatments, symptomatic therapies, and supportive interventions; (ii) Facilitating translational research to bridge the gap between basic science discoveries and clinical applications, accelerating the development of innovative treatments and improving patient care. Several potential reasons have been considered for the limited success of the previous clinical trials, including wide clinical heterogeneity of PD, inadequate definition and documentation of target assignation, lack of appropriate biomarkers, and evaluated outcomes, as well as short duration of sequel. Thus, future trials may be more personalized, trying to select proper participants and therapeutic strategies, investigate combination therapies that would address wider pathogenicity, and assess nonmotor PD features.^359^

## Outlook, Roadblocks, and Perspectives

Continued research efforts hold promise for developing novel treatments, identifying biomarkers for early diagnosis, and understanding disease mechanisms. Personalized treatment approaches based on genetic, biomarker, and clinical profiles may improve outcomes and tailor therapy to individual patient needs. Integration of digital health technologies, wearable devices, and artificial intelligence (AI) could revolutionize disease monitoring, management, and therapeutic interventions.

Emphasis on patient-centered care, shared decision-making, and holistic approaches to treatment may enhance quality of life and well-being for PD patients and their caregivers. Collaboration among researchers, clinicians, patients, advocacy groups, and industry partners is essential for accelerating progress, translating scientific discoveries into clinical applications, and addressing unmet needs. Empowering patients and caregivers through education, support services, and advocacy initiatives can raise awareness, reduce stigma, and drive policy changes to improve access to care and resources.

The main future research priorities need to include: (i) slowing disease progression via integration of precise biomarkers and targeted therapies and (ii) improving the current symptomatic treatments, including surgical and infusion therapies, in an effort to postpone complications and improve durable patient management. Current studies are targeted at α-synuclein aggregation, along with efforts to target specific genetic pathways. In terms of symptomatic treatment, the goal should be to refine existing strategies and promote the advancement of novel therapeutic approaches targeting levodopa-resistant symptoms and clinical indicators that significantly worsen quality of life.^360^

Despite significant research efforts, there are currently no disease-modifying therapies available to slow or halt the progression of PD. Developing effective neuroprotective treatments remains a major challenge. Nonmotor symptoms of PD, including cognitive impairment, psychiatric symptoms, autonomic dysfunction, and sleep disturbances, can be challenging to manage and may significantly impact quality of life. There is variability in treatment response among PD patients, and individualized approaches to therapy are needed to optimize outcomes. Identifying predictors of treatment response and refining precision medicine strategies are ongoing challenges. Disparities in access to specialized care, support services, and research opportunities exist among PD patients, particularly in underserved communities and rural areas. Addressing these disparities is crucial for ensuring equitable care delivery and improving outcomes.

In summary, while there are reasons for optimism regarding advancements in PD research, personalized care approaches, and technological innovations, significant challenges and roadblocks remain. Overcoming these challenges will require sustained efforts, collaboration, and a multifaceted approach to improving the outlook and quality of life for individuals affected by PD.
